# Rapid Detection of Direct Compound Toxicity and Trailing Detection of Indirect Cell Metabolite Toxicity in a 96-Well Fluidic Culture Device for Cell-Based Screening Environments: Tactics in Six Sigma Quality Control Charts

**DOI:** 10.3390/app12062786

**Published:** 2022-03-09

**Authors:** Bob Lubamba, Timothy Jensen, Randall McClelland

**Affiliations:** 1 SciBioix, 6409 Fayetteville Rd., Suite 120#64, Durham, NC 27713, USA; 2 SciKon Innovation, Inc., P.O. Box 9100, Chapel Hill, NC 27515, USA; 3 Azture, Inc., P.O. Box 1759, La Jolla, CA 92038, USA

**Keywords:** predictivity, hepatoxicity, liver, screening, microfluidics, multiwell, *in vitro*, compound toxicity, cell metabolite toxicity, drug discovery

## Abstract

Microfluidic screening tools, *in vitro*, evolve amid varied scientific disciplines. One emergent technique, simultaneously assessing cell toxicity from a primary compound and ensuing cell-generated metabolites (dual-toxicity screening), entails in-line systems having sequentially aligned culture chambers. To explore dual-tox screens, we probe the dissemination of nutrients involving 1-way transport with upstream compound dosing, midstream cascading flows, and downstream cessation. Distribution of flow gives rise to broad concentration ranges of dosing compound (0→IC_compound_100) and wide-ranging concentration ranges of generated cell metabolites (0→IC_metabolites_100). Innately, single-pass unidirectional flow retains 1st pass informative traits across the network, composed of nine interconnected culture wells, preserving both compound and cell-secreted byproducts as data indicators in each adjacent culture chamber. Thereafter, to assess effective compound hepatotoxicity (0→EC_compound_100) and simultaneously classify for cell-metabolite toxicity (0→EC_metabolite_100), we reveal utility by analyzing culture viability against ramping exposures of acetaminophen (APAP) and nefazodone (NEF), compounds of hepatic significance. We then discern metabolite generation with an emphasis on amplification across μchannel multiwell sites. Lastly, using conventional cell functions as indicator tools to assess dual toxicity, we investigate a non-drug induced liver injury (non-DILI) compound and DILI compound. The technology is for predictive evaluations of new compound formulations, new chemical entities (NCE), or drugs that have previously failed testing for unresolved reasons.

## 1. Introduction

New cell culture devices are populating life science and pharmaceutical R&D benches at escalating rates, each with unique features and various functions [1–3]. Efforts in development are driven by the need to improve human-relevant experimental systems earlier in the drug evaluation process. New systems could arguably be termed “microfluidic devices” or “benchtop bioreactors” and include characteristics such as kinetic flows, complex cell mixtures, and 2-dimensional (2D) or 3-dimensional (3D) culture spaces [4]. Already, several investigative teams recognize the need for integrating flow with compound exposure and acknowledge this emerging technology may bolster high-content screening and predictive computational modeling [5–9]. In regard to platform physical parameters, μfluidic configurations commonly include a culture chamber, or multiple linked chambers, nutrient flow, and controllers such as pumps, valves, and fluid transport tubes [10]. While such devices can be skillfully prototyped, the refinement into mass manufacturing of product has tangible engineering constraints. Manufacture limitations often cause the platform’s bio-functionality to fall short of what biologists, toxicologists, and drug development researchers find valuable [11,12]. Hence, during μfluidic device creation, early knowledge transfer from end-users and enduring communication with product fabricators are critical evolving interchanges. End-user objectives align with targeted use-model strategies, needs for lab compatible accessories, and design conditions that prompt development constraints. Engineering considerations may include material properties to avoid adsorption of compounds onto fabricated polymers [13–15], the process of seeding and removing cells for evaluation [3,16], altered techniques to validate assays [17,18], and device compatibility with lab equipment such as plate readers, imagers, and liquid handling systems.

### 1.1. A Fluidic Platform for Dual-Toxicity Screening

**Figure 1.**
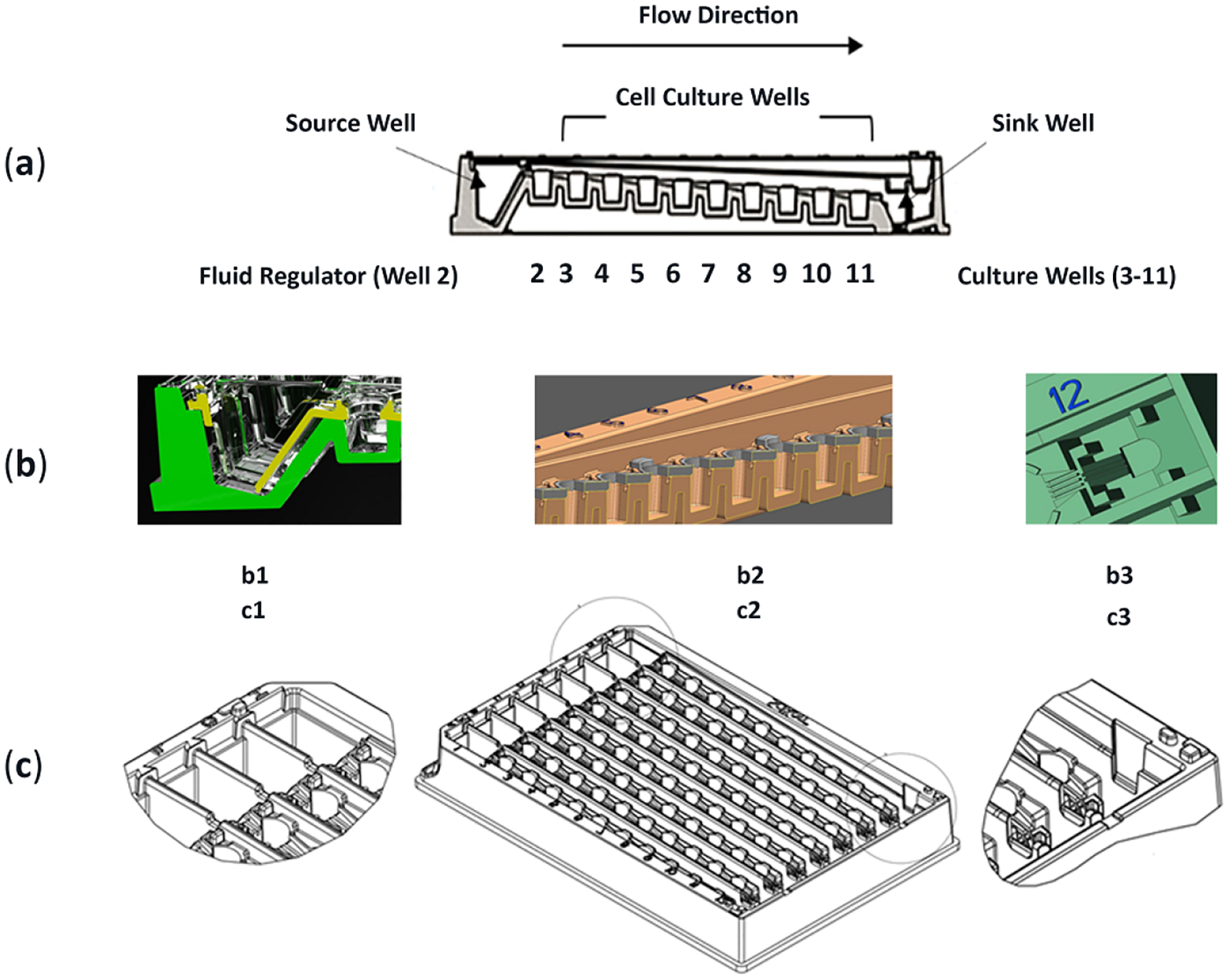
Characteristics of fluidic culture system. (**a**) Profile schematic highlighting source well (well 1), 10 connected compartments (wells 2–11), and sink well (well 12). Wells 2–11 having 0.5 mm decrease in height from each well bottom; total 4 mm height difference. (**b1**) Profile of source well (well 1) and regulator well (well 2; acellular) allowing nutrient media to flow between assembled components (i.e., yellow and green accents). (**b2**) Profile of wells 3–11 with polystyrene polymer overlay (grey) covering microfluidic channels. (**b3**) Top view of sink well (well 12) that is covered by syphon wick (wick not shown). (**c1**) Top CAD view of source well (well 1). (**c2**) Top CAD view of fluidics platform. (**c3**) Top CAD view of sink well (well 12).

In this study, a multiwell μfluidic platform is targeted to monitor cellular effects from both primary drug-toxicant and secondary generated cell byproducts (byproducts defined as one or more metabolites). Regarding static culture systems, past literature discloses fundamental limitations of *in vitro* testing methods that lack fluid flow to indicate no chemical gradients, no metabolite distinctions, and no signaling through interconnected compartments [19]. This no-flow technique can result in data that is less predictive of how a compound will respond, or be responded to, in the *in vivo* environment. Associatively, even *in vivo* animal testing can be unpredictable of human physiology due to phenotypic differences in cell types and organ systems [20–22]. Strategically, benchtop μfluidic environments that better mimic *in vivo* flow networks are gaining research and development interests [3,12]. In this context, we stage the SsWaterfall fluidic culture system (Figure 1), an exposure platform having two unique traits, (1) unidirectionality with non-recirculating fluid flows, and (2) the sequential assembly-line of cell cultures. The unidirectional fluid flow is configured as 1-way transport and conceptualized as a waterfall stream (fluidics) trickle flow (time-influential), having riverbeds positioned all along its downstream path (culture spaces). The non-recirculation feature infers that drug distributions and generated cell secretions remain as distinguishable data indicators, unblended across location and time, as sample traits that are critical descriptors for definitive evaluations amid culture spaces. Comparatively, most precedent μfluidic designs innately mix their nutrient elements (recirculating systems) and inherently blend sample distinctions that inhibit definitive cell function analysis amid adjacent culture spaces. The sequential assembly-line of cell cultures can be envisioned as a production line (as in manufacturing) that is arranged so a product (dose of the compound) is moved sequentially along the assembly line (μfluidic channel) and across workspaces (culture wells). Herein, a procedure is performed at each location (cell function/well) prior to moving refined products (cell signals) to the next downstream location (adjacent culture well). Paraphrased, upstream compound dosing and upstream cell responses influence downstream culture wells. Irrespective of flow path or generated cell byproduct, the μfluidic system allows for modular tissue arrangements with passive fluid transport that is regulated by hydrodynamics, capillary action, and a downstream syphon to pull media across culture chambers (i.e., no pumps, no valves, and no tubes [23]). The flow methodology and approach are essential for studying microphysiological system (MPS) arrangements for predictable compound efficacy and toxicity.

### 1.2. The Organ of Investigation Is Liver with Cell Culture Being Hepatoctyes

Drug failure during *in vivo* clinical trials or the retraction of a drug from an already available commercial compound can be correlated with a lack of effective *in vitro* model systems; namely, culture platforms that lack integration of flow dynamics and lack complex cell interfacing as naturally observed amid native tissues [24]. It continues to be shown that many clinical failures are due to unpredictable effects and unforeseen hepatotoxicity [25–27]. As one model template, DILI is responsible for many post-market drug withdrawals in the European Union [28] and the United States [29,30]. *In vivo*, certain hepatic toxicities occur as the native liver generates cell metabolites that can cause liver damage [31]. To wit, the advancement of *in vitro* models that better mimic behaviors of native liver tissue remains an industry challenge, with a focus on improved efficacy and decreased toxicity [32]. To enhance scientific understanding and development, the *in vivo* organization of cells in the liver implies that *in vitro* culture systems might benefit from cell–cell communication and intrinsic interactions between a parent compound and generated cell-byproducts. Amid select drug subgroups, literature already discloses that stagnant (i.e., no-flow) hepatic culture systems do have a limited capacity to predict facets of toxicity or efficacy outcomes [33–35]. In efforts to broaden the success of predictive studies, a major focus of this research was to demonstrate the ability of a stand-alone and disposable μfluidic platform to simultaneously detect compound toxicity (upstream) and cell-metabolite toxicity (downstream) in one integrated system. To evaluate cell-response modulations, due to perpetual shifts of ascending exposures, two stable but different hepatic cell models were exploited; the human HepaRG cell-line (Biopredic International, Saint Gregoire, France) and freshly isolated rat hepatocytes (Lonza, Basel Switzerland—Durham, NC, USA location).

### 1.3. Exposure Compounds and Cell Health Readouts

Hepatocyte cultures were exposed to diclofenac, APAP, aspirin (ASA), NEF, and dimethyl sulfoxide (DMSO). Diclofenac is a known cytochrome P450 3A4 (CYP3A4) inducer. APAP is a well-studied standard with known metabolites APAP-glucuronide (APAP-Glu), APAP-sulfate, and APAP-glutathione (APAP-GSH). ASA and NEF are non-DILI or DILI compounds, with DMSO being the compound control vehicle [36]. To appraise cell culture health, cell function readouts include lactate dehydrogenase (LDH) activity, CYP3A4 inducible-fold levels, glutathione (GSH-assay), and live/dead cell image responses (DAPI, calcein AM, CellTox Green, ethidium homodimer). To appraise metabolite generation, liquid chromatography–mass spectrometry (LC/MS) was used to assess supernatant samples.

## 2. Materials and Methods

### 2.1. Device, Design, Material, Construction, Culture Surface, and Attributes

#### 2.1.1. Device

Each fluidic culture system consists of 96 wells, 8-row replicate rows (A-H), with wells connected by embedded micro-pathways that link 12 wells across a row (Figure 1). Compound dosing occurs in well 1 (upstream), then the system auto-generates unidirectional flow left-to-right (well 1→12) by hydrodynamics, capillarity, and wicking, i.e., stair-step waterfall design without external pumps. The adjustable flow rate is regulated by removing medium from well 12 (Sink well) and adding medium + drug into well 1 (source or dose well). The frequency and volume of the dosing compound controls the rate by which the drug migrates across the row [23].

##### Structure and Lab Compatibility

The platform is based on the Society for Biomolecular Screening (SBS) parameters and formatted as a 1/2 area 96-well plate definition for use in existing scientific infrastructures to include plate readers, imaging systems, and pipettors, i.e., designed lab-compatible, device manageable, with user familiarity, Table 1. The device is considered a medium-throughput system that has reduced cost structures based on decreased cell quantities (1/2 area 96-well), a reduction in nutrient volumes (100 μL/well), and lower amounts of drug/compound for exposure findings (scaled-down μchannels).

#### 2.1.2. Material and Cell Compatibility

The device is manufactured from laboratory approved traditional polystyrene, suitable due to its already established quality control and history as a validated cell culture surface material with known assay outcomes. Still, because manufactures of polymers have variant constituent formulas and resin adaptations, non-specific binding should be appraised for quality control validation [23]. Moreover, the optical transparency property of polystyrene enables ease of cell observation and imaging.

##### Mass Production for Repeatability

The microfluidic biotool is suited to be a cost-effective tissue culture system that can be injection molded for mass production, construction repeatability, and dosing/exposure applicability. The two separate injection molded pieces are ultrasound welded together (Figure 1b); polystyrene welds are preferred as the process avoids extraneous adhesive or diverse material contaminants within the culture areas.

##### Culture Surface and Treatment

The device’s polystyrene surfaces have been transformed into tissue culture plastic (TCP) by corona plasma treatment. TCP adjusts surface tensions to aid in wetting of a solid by a liquid and is utilized as a surface foundation to support numerous adherent cell types for cell plateability and culture stability [23]. The dyne levels of untreated polystyrene (34 dyne/cm^2^) and TCP (43 dyne/cm^2^) were measured to quantify surface tension. EnerDyne^™^ pens (Enercon; Menomonee Falls, WI, USA) ranging from 30 to 66 provided a means of quantifying the contact angle, θ, which is defined geometrically as the angle formed by a liquid at the three-phase boundary where a liquid, gas, and solid interact.

#### 2.1.3. Assembly of μChannels

The platform has two assembled components. The base unit (96-well fitted plate) has open pathways connecting each well within a row. Atop the base unit are fused row covers over each row, forming a ceiling and two sides of each micro-channel. A computer aid design (CAD) illustration of aligned culture wells shows the intersection between the row-cover, base unit, and the formed fluidic-channel which connects every adjacent well (Figure 1b). Wells in column 1 and 12, i.e., fluid source and sink, are different from well sites in columns 2–11, having discrete functionality described in Table 1.

### 2.2. Device Preparation for Cell Seeding (Static) and μFluidic Operation (Flow)

#### 2.2.1. Prepare Platform for Cell Seeding Using No Flow Conditions (Static)

Humidify the system by placing it in a humidified incubator for 2 h or longer (overnight). The pre-incubation aids in plate wettability for small-area cell cultures and also diminishes fluid capillary climb along the cell-chamber sidewalls. The inhibition of capillary climb is important to retain disconnected “no-flow” culture traits. Seed the cells in 50 μL of media/well (cell density is cell type dependent). The 50 μL volume is beneath the entrenched micro-channels. Fill the wells from right to left (uphill): column 11, then 10, then 9, 8, 7, 6, 5, 4, and 3; column 2 is normally left acellular as it is in very rapid equilibrium with the source well. Allow cells to settle and attach before initiating fluidics; seed timelines are dependent on cell type.

#### 2.2.2. Connecting Culture Wells and Initiating Fluidic Conditions (Flow)

First, remove the spent media from the cell seeding and acclimation process. Next, add 350 μL of fresh media to the sink well (well 12), 100 μL of fresh media into columns 2–11, filling the wells from right to left (Column 11, 10, 9 … 2), and 400 μL of fresh media into the source well. Return the filled platform to a humidified 37 °C incubator, flow begins automatically. The same process is followed regardless of whether it is a cell line, primary cells, or different cell phenotypes seeded in separate multiwell chambers within the device.

### 2.3. Cell Seeding Conditions in μFluidic Device

HepaRG cells and Rat hepatocytes were cultured in the platform. Cell counts were performed using a hemocytometer and both phenotypes were seeded at 50,000 cells/well in 50 μL of media (no flow conditions); i.e., 312,500 cells/cm^2^. All cell cultures were maintained in a 37 °C incubator at 5% CO_2_. All culture material were purchased from GIBCO, Waltham, MA, USA unless otherwise stated.

#### 2.3.1. Human HepaRG Cell Line

The μfluidic platform’s tissue culture surface was suitable for seeding and long-term culture [37,38]. Cells were grown in manufacture recommended media, Williams’ Medium E (Life Technology, Carlsbad, CA, USA) containing supplements for growth (Biopredic). HepaRGs were differentiated before procurement, not passaged, and required a 7-day acclimation/maturity period before treatment. Throughout acclimation, the medium was renewed every 3 days in no-flow static conditions. On day 3, cells reached contact inhibition. On day 8, all media were removed, then the sink well was filled with 350 μL of media, wells 2–11 filled with 100 μL of media, and the source well maximized at 500 μL (400 μL media + 100 μL of treatment compound).

#### 2.3.2. Rat Hepatocytes Freshly Isolated

Prior to cell seeding, rat tail collagen (Sigma-Aldrich, St. Louis, MO, USA) was diluted into cold cell culture medium to 0.05 mg/mL and 30 μL (1.5 μg/well) was applied to each culture well of the device, allowed to attach for 1-h at 37 °C, and washed with rat hepatocyte culture medium containing high-glucose Dulbecco’s modified Eagle’s medium (DMEM) supplemented with 5% fetal bovine serum (FBS), 2 mM Glutamax, 100 U/mL penicillin, and 10 μg/mL streptomycin. For seeding, cells require a 24-h acclimation period before treatment. During seeding/acclimation, medium renewal is not needed during no-flow static conditions. On day 2, all media was removed, then the sink well was filled with 350 μL of media, wells 2–11 filled with 100 μL of media, and the source well maximized at 500 μL (400 μL media + 100 μL of treatment compound).

### 2.4. Drug Treatment during Cell Culture

In the fluidic platform, compounds were added to the source well (Figure 1a). Daily, 100 μL of media was removed from the last column (well 12), and 100 μL of fresh media containing vehicle (0.1% DMSO) or drug was added to the source well every 24 h. The compounds evaluated for cytotoxicity, *in vitro*, were assigned to one of 2 categories, non-DILI and DILI, using information extracted from the peer-reviewed scientific literature [39,40] and data contained in product labels. APAP, ASA, and NEF were purchased from SigmaAldrich (Burlington, MA, USA). Cells were washed with phosphate-buffered saline (PBS) and exposed to 0.1%DMSO (vehicle-control) medium containing the desired concentration of 1 mM APAP, 25 μM ASA, and 16 μM NEF for 7 days.

### 2.5. Suitability of FITC Surrogate Drug to Ascertain Compound Concentrations

The fluorescent tracer fluorescein (FITC) was used as a surrogate evaluator (i.e., fluid tracer) for the drug dissemination. FITC, dissolved in the medium at 5× desired concentration (5 × 1 μM), was loaded into the source well adjacent to the actual drug compound row, exactly the same as dosing and in the same volume and frequency as compound re-dosing (100 μL every 24 h), unless otherwise noted. Prior to each dosing/feeding, the fluorescence was measured in each well (CLARIOstar microplate reader, BMG LABTECH USA, Cary, NC, USA) at ex485/em525 including the z-height offsets from Figure 1 and Table 1, with gain optimized for 1 μM FITC. A standard curve of FITC fluorescent was used to determine the shift of FITC concentrations (0–1 μM) across the device. Replicate fluorescent signals across the device, wells 3–11 (n = 9), were averaged and plotted against known concentrations.

### 2.6. Quantification of APAP Drug Metabolites

Supernatants were collected after 4 h, 24 h, 48 h, and 72 h of incubation and stored at −80 °C until quantification. The addition of acetonitrile precipitated samples for drug metabolite analysis. After centrifugation, the supernatants (containing 90% acetonitrile) were analyzed by LC/MS (OpANS, Research Triangle Park, NC, USA).

### 2.7. Assays to Evaluate Modulations in Cell Health

#### 2.7.1. Live and Dead Assays (Imaging)

Cells were stained with a mixture of 1 μg/mL of Hoechst stain (Sigma-Aldrich, St. Louis, MO, USA), calcein-AM (2 μM) and ethidium homodimer (EthD)-III (5 μM) purchased from (Thermo Fisher, San Diego, CA, USA), or, with a mixture of 1 μg/mL of Hoechst stain (Sigma-Aldrich, St. Louis, MO, USA) with 1:1000 CellTox Green Stock solution (Promega, Madison, WI, USA), followed by 1× exposure for real-time assay multiplexing. Fluorescently stained cells were auto-imaged with high-content imaging (Cytation 5 Reader/Imager, BioTek, Winooski, VT, USA), using objective software allocating for x, y, and z-height stair-step differences.

#### 2.7.2. LDH Assay

LDH release was measured with a commercially available LDH assay kit (Cytotoxicity Detection Kit, Roche, supplied by Fisher Scientific, Waltman, MA, USA) requiring flow stoppage, incubation period, and generation of discrete data. Briefly, LDH, which becomes released in the cell surrounding environment, causes a reduction in NAD+ to NADH and H+ through the oxidation of lactate to pyruvate. After that, a catalyst (diaphorase) transfers H/H+ from NADH + H+ to a tetrazolium salt (iodonitrotetrazolium, INT) to form a redcolored formazan salt. The amount of color produced is then colorimetrically measured at a wavelength of 490 nm by a spectrophotometer. The colorimetric was measured at room temperature on a BMG CLARIOstar plate reader (BMG Labtech, Cary, NC, USA). LDH content was shown as fold change over vehicle controls.

#### 2.7.3. CYP3A4 Luminescence Assay

CYP3A4 enzyme activity was determined using the P450-Glo CYP3A4 assay (V9001), which contained the substrate luciferin isopropyl acetal (luciferin-IPA) and luciferin detection reagent (Promega, Madison, WI, USA) requiring flow stoppage, incubation period, and generation of discrete data. Briefly, after exposure to compounds, cells were washed with PBS and incubated at 37 °C with luminogenic CYP substrate dissolved in medium without phenol red. After 30 min, the medium was transferred to a 96-well opaque white luminometer plate, and the same volume of luciferin detection reagent was added for 20 min. Luminescence was measured on a BMG CLARIOstar plate reader (BMG Labtech, Cary, NC, USA). CYP3A4 enzyme activity was calculated as fold change over vehicle controls.

#### 2.7.4. Glutathione (GSH) Level

GSH activity was measured with a commercially available GSH assay kit according to the manufacturer’s instructions (Promega) requiring flow stoppage, incubation period, and generation of discrete data. Briefly, after treatment with compounds, cells were washed with PBS, and 30 μL of GSH-Glo reagent 1X with luciferin-NT substrate and glutathione S-transferase were added in each well. After 30 min, the lysed cells were transferred in a 96-well opaque white luminometer plate and added the same volume of reconstituted luciferin detection reagent for 20 min. GSH activity was measured using a luminometer. This assay is specific for GSH; other thiols do not cause interference in the assay. Cellular GSH concentration was calculated as fold change over vehicle controls.

### 2.8. Statistical Analysis and Tactics for Lean Six Sigma Control Charts

**Figure 2.**
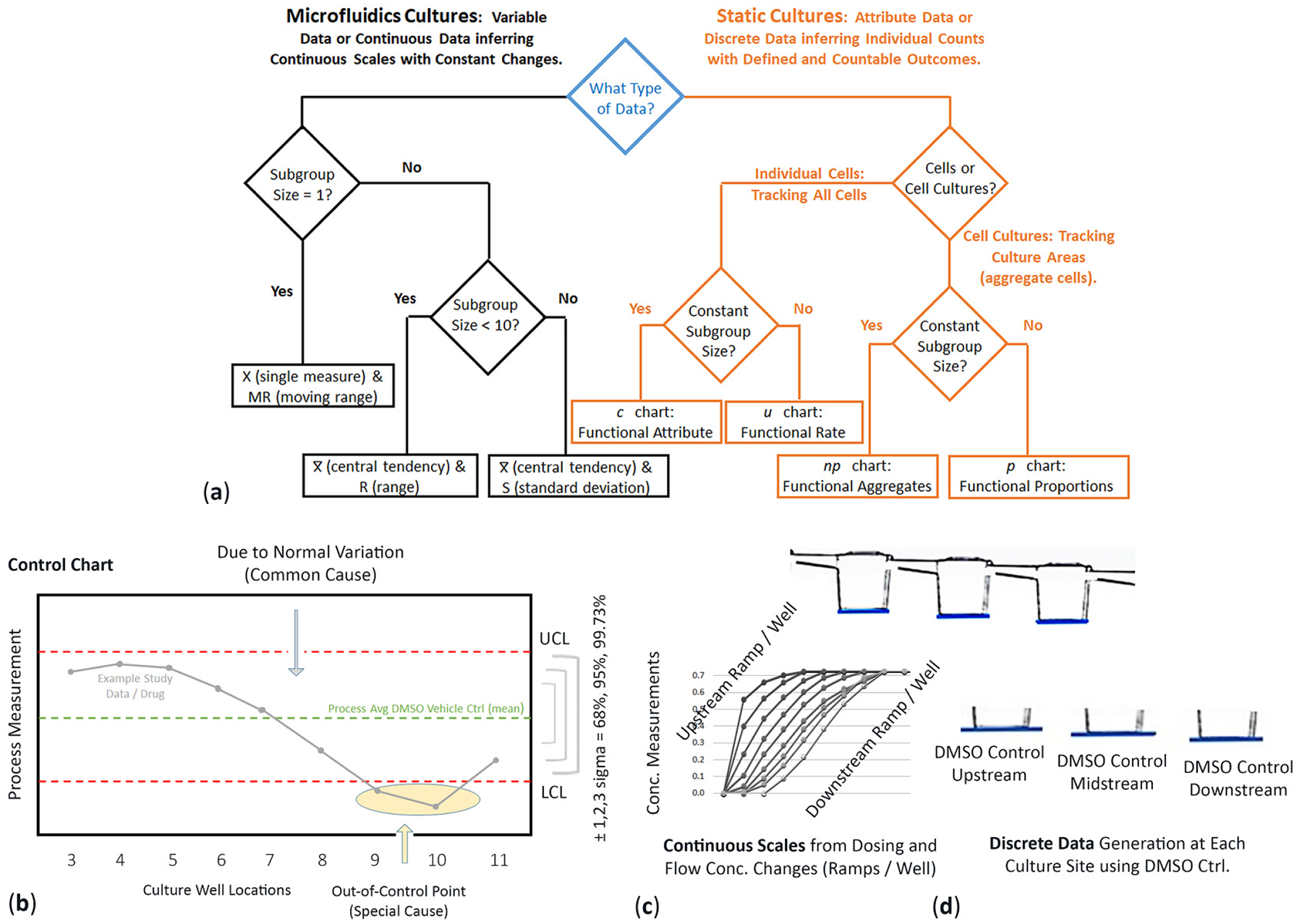
(**a**) Logic tree diagram to select statistical control charts based on data interpretation for microfluidic vs. static cultures. Data types correlate with appropriate statistical analysis. (**b**) X and MR control chart. Variability is described by Nelson rules and tests for special causes or unusual patterns on a production line. (**c**) Continuous exposure scales experienced in upstream wells, midstream wells, and downstream wells. (**d**) DMSO controls are different at each multiwell site along the channel.

Because different techniques are needed to evaluate the change in platform environments (drug exposure vs. cell assay), statistical relevance is presented in two formats, either continuous variable or discrete variable outcomes [41,42]. Selection is based on a logic tree diagram to indicate appropriate data charts (e.g., X and MR, *u* or *p* charts, etc.) that are aligned with lean six-sigma practices (Figure 2a). The tree diagram is adapted from traditional manufacture production lines (originally tracking production line errors) but reformatted into cell-culture features that correlate with μfluidic or static cell-culture schema. Continuous variable is defined as the infinite number of possible measured values as it is impossible to list all scenarios (e.g., variable changes in cell functions). Discrete variable is defined as data that is countable that enables a set number of possible values (e.g., number of cells). Charts are equally valuable.

#### 2.8.1. Continuous Data for μFluidic Culture

Complex numbers and varying data that are measured over a specific interval (range), such as time or concentration levels. Values are simply not countable but require detailed measurements that can have separate outcomes at any given point, referring to unspecified numbers of possible measurements between two realistic points. The numbers are not always clean and tidy (e.g., curves and skews). Continuous data is about accuracy and typically involves fluctuating numbers between two presumed points. For continuous μfluidics, control charts are used to determine if cell responses are in states of statistical control (predictable) or have become unstable (i.e., variation with unusual outcome such as cell death or generated cell metabolites); Figure 2b. In this study, control charts are used to analyze cell performances across μfluidic culture wells 3–11, conceptualized as a production assembly line, each well with functional responsibility prior moving product (cell signals) to the next location. DMSO vehicle control data is used to signify process average (mean) and significance offsets ±1σ (68%), ±2σ (95%), and ±3σ (99.73%). For normal distribution rules, study data is expected within standard deviation variances [42,43]. If data falls outside of significant offsets, variations imply special or assignable causes, e.g., Nelson rules [44], such as inducible cell functions addressing injury vs. non-injury, compound weed-out, data oscillations, pre-screening before study, and operator interventions. Terminology to define cell modulation traits involving process shifts, special causes, and unusual outcomes are further described in Appendices A.1 and A.2.

#### 2.8.2. Discrete Data

Numerical data includes whole and concrete numbers with specific fixed data values remaining constant over specific time intervals. Synonyms include disconnected, separate, and distinct. Biological replicates from three separate wells were averaged to obtain the mean and standard error of the mean for each treatment dose. To evaluate if a drug induced cellular changes, the comparisons were made between compounds and vehicle controls. Statistical analysis was performed using a two-way ANOVA test to assess the significance of responses, * *p* < 0.05, ** *p* < 0.01, *** *p* < 0.001, and **** *p* < 0.0001; ns: not significant. GraphPad Prism software (GraphPad, San Diego, CA, USA) was used to generate all graphs.

### 2.9. Standard Operating Procedure (SOP) for One Drug in One Fluidic Culture System

**Figure 3.**
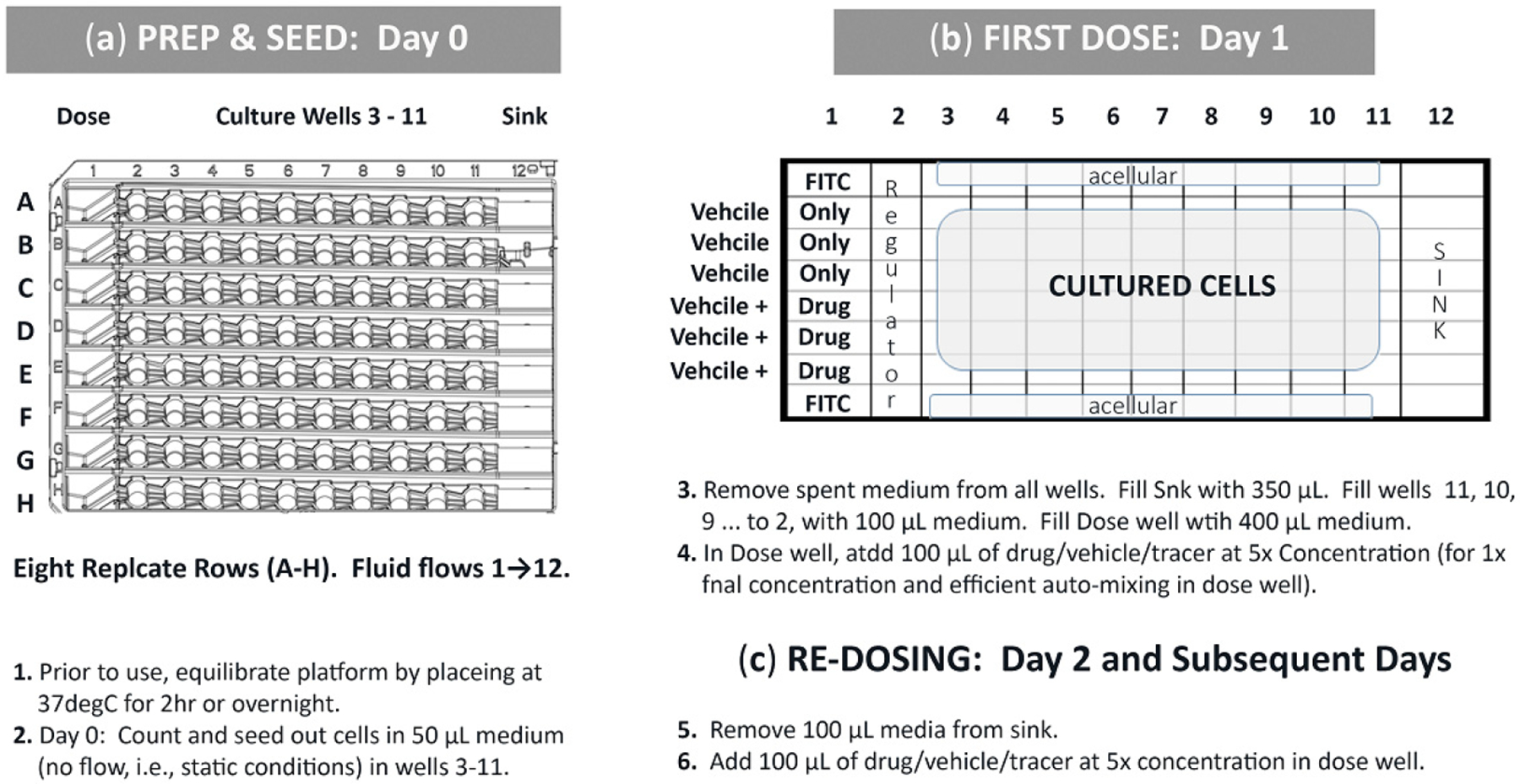
Standard operating procedure (SOP) for 1-drug study (n = 3). (**a**) Preparation and cell seeding. (**b**) First dose into well 1. (**c**) Re-dosing into well 1.

One fluidic device has 8 replicate rows (A-H), Figure 3, affording statistical relevance. For a 1-drug study being assessed using one device (Figure 3b), two rows remain acellular and are used for FITC fluid flow tracers (n = 2; A and H); three cellular rows for DMSO vehicle only control (n = 3; B, C, D) and three cellular rows for DMSO vehicle + study drug (n = 3; E, F, G). A cursory protocol is presented to delineate experiment preparation to convey cell seeding, first compound dose, and compound re-dosing (Figure 2, items 1–6). Briefly, cells are seeded in stagnant conditions and remain in no-flow status while cells acclimate. To begin flow, surplus nutrient media is added to wholly fill culture wells, automatically filling μchannels, and intrinsically connecting well-to-well fluidic pathways such that a nutrient stream is formed across culture wells 1→12. Compound dosing is initiated in well 1 (bolus input; 100 μL). The compound then auto-dilutes while flowing from well 1 and towards well 12. Well 2, being the peak inflection point between uphill nutrient flow energies (wells 1-to-2) and downhill flow forces (wells 2–12), remains acellular and operates as a fluid regulator. The first cellular well, well 3, is exposed to compound concentrations at higher/faster rates than downstream wells. The last cellular well, well 11, experiences the lowest/slowest compound concentrations with delayed exposures. If one entire row is considered as a single unit, i.e., not 12 sequentially linked culture sites, then the bulk flow rate approximates 100 μL/24 h (4.16 μL/h = 0.069 μL/min) and corresponds to hydrodynamic forces (0–1 h), transitional forces (1–3.3 h), and sustained equilibrium forces (3.3–24 h @ re-dose) [23].

## 3. Results

### 3.1. Operational Insights for Compound Dosing, Quantified Concentration Gradients, Flow Distribution Patterns, and Transforming Well-to-Well Exposures

**Figure 4.**
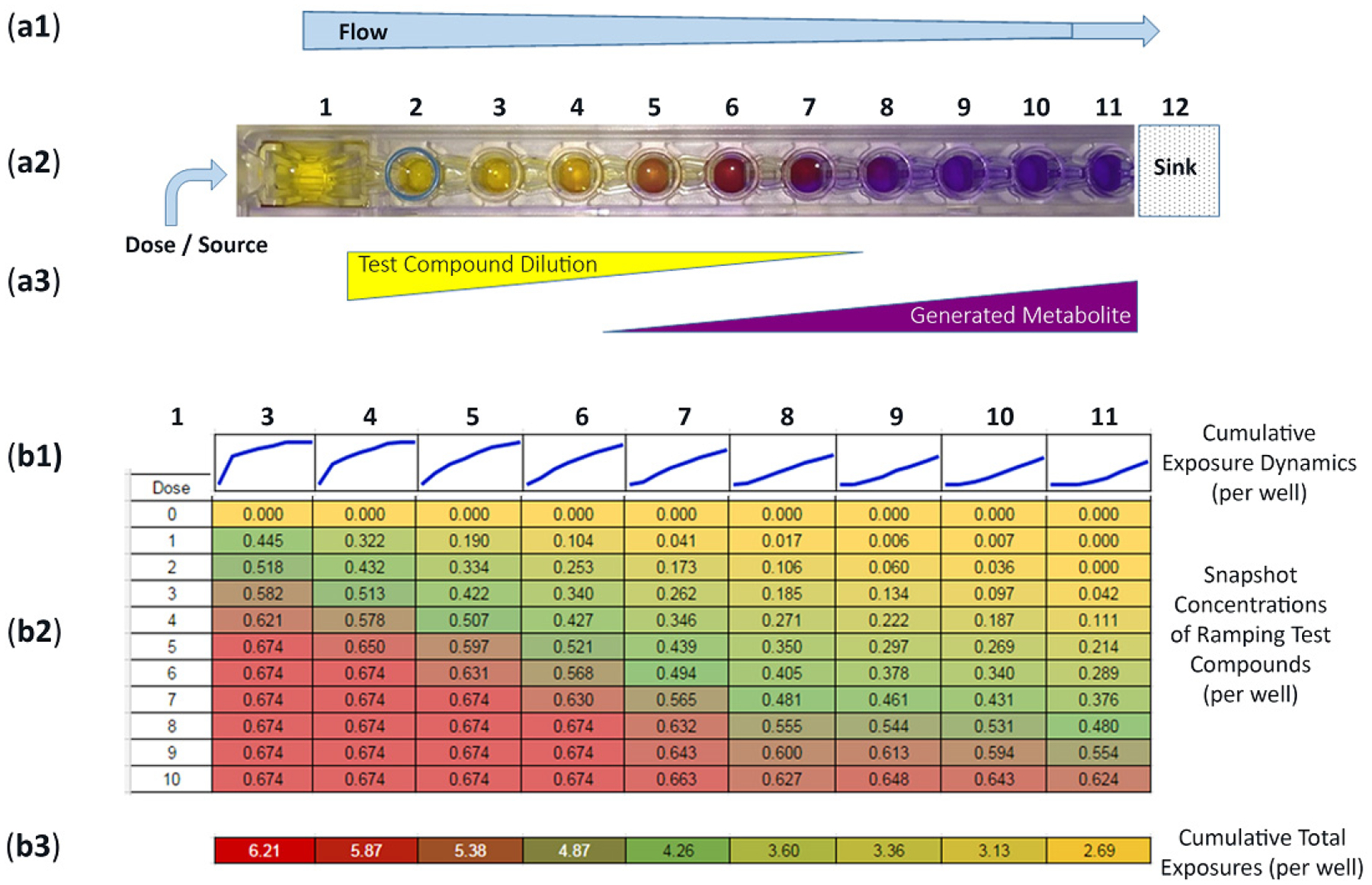
Operational insights. (**a**) Top view simulation of one microfluidic channel signifying dual exposure gradients of test compound (dosed into well 1) and cell generated metabolite. (**b**) Modulating experimental exposures of test compound throughout one device row having ten (10) re-doses of 100 μL over 3 days. Values 0→1 equate to 0→100% of dose. Dose “0” is culture media without compound (i.e., no-dose baseline). (**b1**) Linescans displaying a timeline of aggregate exposure kinetics, per well, with compound exposure traits ranging from asymptotic (wells 3, 4), approximately linear (wells 7, 8), and delayed increase (lag of exposures in wells 10, 11). Subsequently, fully developed linescans progress into natural logarithm, linear, and delayed exponential growth [23]. (**b2**) Well-specific, data centric, cumulative exposures over time (i.e., area under curve); similar to a radiation model. The final equilibrium drug concentration is very similar across the entire plate (dose 10; wells 3–11; 67.4→62.4%). (**b3**) Total “Cumulative Exposure” is very different in each well, 6.21→2.69.

To quantify fluid flow patterns, we surveyed a single row (1-of-8) over 10 consecutive dosing periods (Figure 4(a1,b2)). Acellular wells are well 1 (source/dose), well 2 (fluid regulator), and well 12 (sink/wick). Cell cultures being wells 3–11. For cell seeding and μchannel flow preparation, the entire row is filled with shared nutrient media (e.g., uncolored and clear). When a bolus drug is placed into well 1, the drug flows toward well 12, creating a dilution gradient of compound flow from high-to-low concentrations (Figure 4(a1–a3), compound dilutions). Then, if cell metabolites are generated in downstream culture chambers, these cell byproducts are pushed towards well 12, hypothetically creating low-to-high concentrations (Figure 4(a2,a3), generated metabolites). To track parent drug exposures and quantify real-time shifts of concentrations, FITC is used as a drug surrogate with FITC concentration gradients monitored/quantified using a microplate reader, ex485/em525 [23]. The Data Table in Figure 4(b2) shows that 1 μM FITC is dosed into the source well (well 1) on 10 chronological iterations. Dose “0” is before FITC dosing (i.e., baseline media) measuring the common and shared nutrient media that is identical across the entire row, wells 3–11, quantified “0.000”. Dose 1 shows well 3 at 0.445 (44.5% of 1 μM FITC dose), well 4 at 0.322 (32.2%), and well 11 at 0.00 (0%), signifying an initial gradient of dose-flow from wells 3–11. A 5th dose displays well 3 at 0.674 (67.4% of 1 μM FITC dose) and well 4 at 0.650 (65.0%) and well 11 at 0.214 (21.4%). A 10th dose displays well 3 at 0.674 (67.4% of 1 μM FITC dose), well 4 at 0.674 (67.4%), and well 11 at 0.624 (62.4%). The distribution of FITC increases after each dose, higher from left-to-right. Quantified data can be assessed across one entire row for wells 3–11 (Figure 4(b2); horizontal display) or within 1 culture well over time periods (Figure 4(b2); vertical display). For individual culture wells (vertical display), time-dependent exposures are portrayed as escalating linescans, i.e., flow timelines/well (Figure 4(b1)). During the 10-dose study, well 3 experiences the quickest/highest exposure (i.e., asymptotic with steepest slope), well 7 depicts an approximate linear exposure pattern, and well 11 foretells a delayed exposure dynamic (i.e., delayed increase). Subsequently, fully developed linescans progress into natural logarithm, linear, and delayed exponential growth [23]. Associatively, cumulative well exposures are depicted in Figure 4(b3). Accrued exposures are similar to a radiation exposure model with area-under-the-curve (AUC) quantifications. Well 3 has the highest AUC at 6.21, wells 7 and 8 with midrange values of 4.26 and 3.60, and well 11 being the lowest at 2.69. From this single-row study, Figure 4(b2), ninety-nine (99) datapoints are generated to establish a trending assessment of non-linear compound concentrations over time and location. From this dosing study, by way of extrapolation, one device equates to 8 rows × 99 = 792 exploitable datapoints.

### 3.2. Incentive for Using FITC Surrogate to Track Actual Compound Distributions across Device Wells 3–11

**Figure 5.**
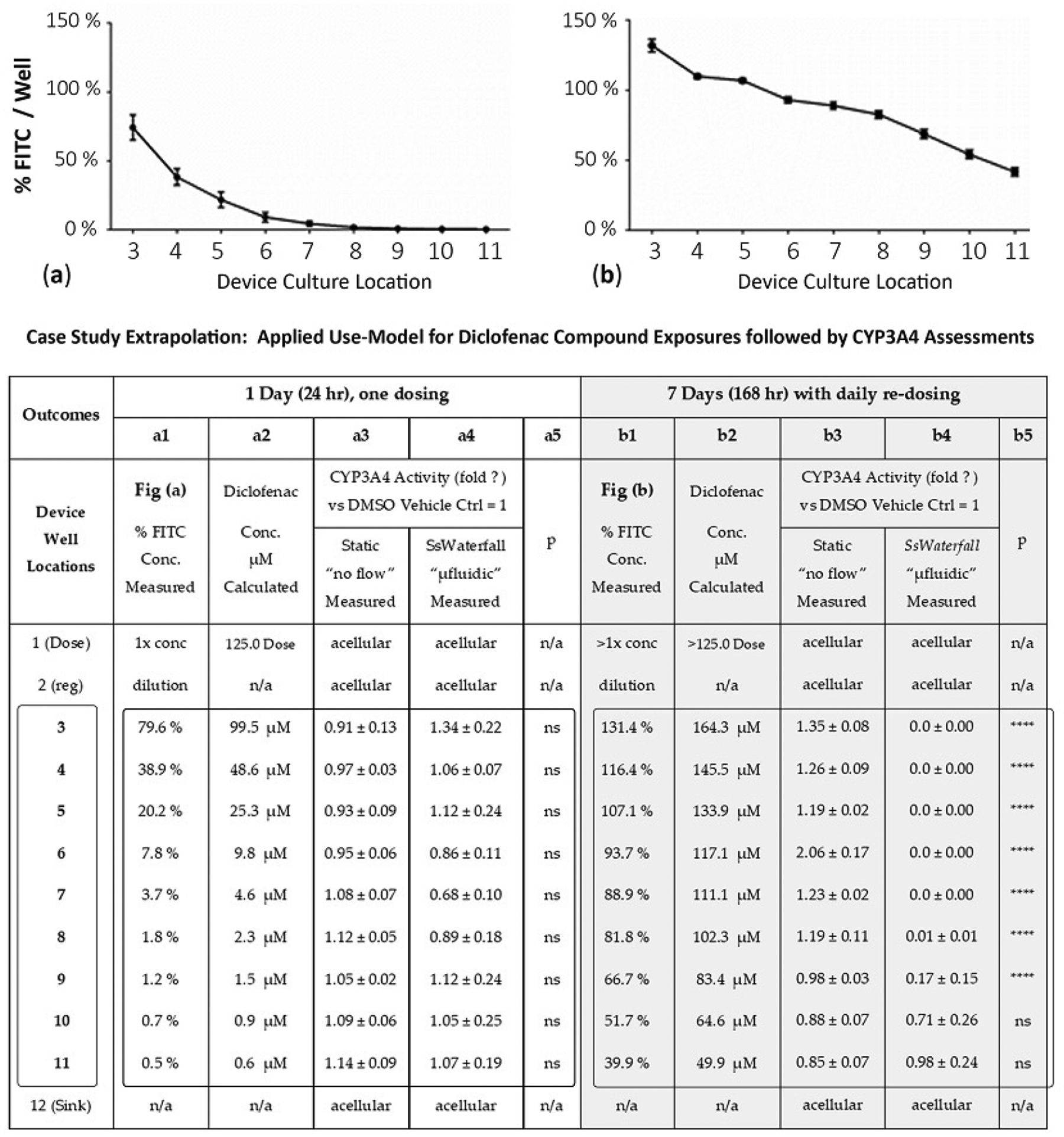
Percentage and quantification of FITC-flow and drug-concentrations in multiwell device. (**a**) FITC concentrations after 1 day, i.e., 24 h, and a single FITC bolus dose into well 1. (**b**) FITC concentrations after 7 days, i.e., 168 h, and daily FITC dosing into well 1. **(TABLE: case study using drug diclofenac):** Comparison of CYP3A4 activity after diclofenac treatment in HepaRG cells. (**a1**,**b1**) % concentrations of FITC surrogate, per well, measured. (**a2**,**b2**) μMolar concentrations of diclofenac, per well, calculated from FITC % (i.e., diclofenac dose (well 1) × FITC % (well specific). (**a3**,**b3**) CYP3A4 fold-Δ using device in “no flow” static mode. (**a4**,**b4**) CYP3A4 fold-Δ from compound dosing in device μfluidics. (**a5**,**b5**) Values are mean ± SEM, (n = 10) **** *p* < 0.00001.

To ascertain actual study compound concentrations across the wells, i.e., spatial and time distributions, 1 μM FITC surrogate was added into a source well in a row parallel to the compound dosing row, simulating an actual compound dose of volume and frequency (drug study). The FITC relative fluorescence units (RFU) are used as a surrogate evaluator in drug dissemination. The fluorescence FITC was measured to estimate drug concentrations after 1 day following a single bolus dose in well 1 (Figure 5a) and after 7 days with daily dosing in well 1 (Figure 5b). In kinetic displays, data curves show the % FITC on the *y*-axis (i.e., % of 1 μM FITC signal) versus device well-sites on the *x*-axis. The relationship aligns time-resolved exposure gradients with % FITC surrogate, used to approximate % drug/well [23]. At 1 Day (24 h), culture well 3 approaches 79.6% of initial dose, well 5 (20.2%), well 7 (3.7%), and well 11 (0.5%). Similarly, at 7 days (168 h), well 3 approaches 131.4% of initial dose, well 5 (107.1%), well 7 (88.9%), and well 11 (39.9%). The dose values > 100% (Figure 5b) are a function of repeat dosing and residual FITC in wells that accumulate in value. These data show how the platform can be used to generate trackable and repeatable FITC gradient concentrations across the μfluidic system. In this, it is acknowledged that molecular weight differences between FITC and other drug categories will shift gradients of flowing exposure dynamics; an awareness that trending line-shifts can be calibrated for predictive accuracy [23].

### 3.3. Validation of Tempered DMSO Compound as an Effective Control Vehicle Using High-Content Cell Imaging across the Sequential Assembly-Line of μFluidic Culture Sites

**Figure 6.**
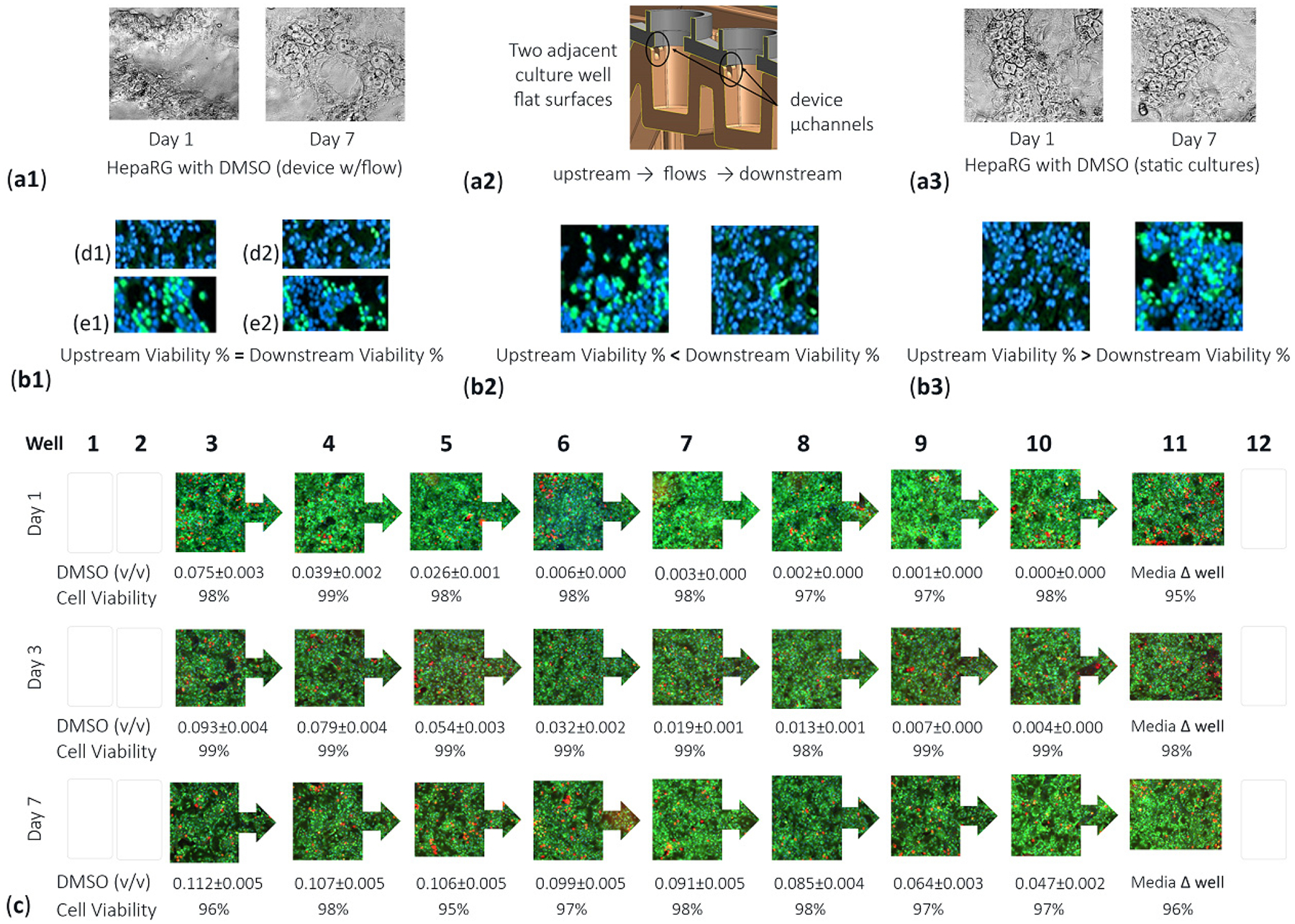
Morphology and viability of HepaRG cells in device multiwell culture locations, post 0.1% DMSO vehicle compound treatment. (**a2**) Profile of device displaying two adjacent wells, two flat culture surfaces, and μchannels entering wells. (**a1**) Kinetic (fluid flow) cultures with cell images at days 1 and 7, phase contrast brightfield 10×. (**a3**) Comparative cell images at days 1 and 7 in static (no-flow) cultures, 10×. (**b1**–**b3**) Fluorescent image captures of cell nuclei stains (DAPI; blue) and non-viable stains (CellTox Green; green) for adjacent device wells, 4× pictorials. (**b1**) Equivalent high cell viability (**d1**,**d2**), or equivalent lower cell viability (**e1**,**e2**). (**b2**) Upstream device well displaying lower cell viability vs. downstream well. (**b3**) Upstream device well displaying higher cell viability vs. downstream well. (**c**) Fluorescent image captures of cell nuclei stains (DAPI; blue), viable cells (Calcein AM; green), and non-viable cells (Ethidium Homodimer; red) to assess variance of cell viability across device wells 3–11. (Day 1) Auto-DMSO concentration gradients after a single 0.1% DMSO bolus dose (0.1 *v/v*) into well 1. (Day 3) After three 0.1% DMSO re-doses into well 1; daily re-dosing. (Day 7) After seven 0.1% DMSO re-doses into well 1. Overall, cell viability remains effectively unchanged, 95–99%.

To investigate effects of the DMSO control vehicle on the viability of cells, HepaRGs were seeded in the μfluidic platform and exposed to the DMSO vehicle for 7 days. HepaRG morphology was evaluated for cells experiencing fluid flow and for cells in no-flow static protocols (Figure 6(a1,a3)). Cell structures were morphologically unchanged with areas of tightly/loosely packed cell densities achieving 100% culture confluency. Yet, given that upstream culture wells feed into downstream well sites, as shown in Figure 6(a2), a variance in cell viability could occur amid adjacent multiwells. Adjacent wells might show cell viability with equivalent high or low measures (Figure 6(b1,d1/d2 or e1/e2)). Adjacent wells might show upstream wells with lower cell viability (Figure 6(b2)). Adjacent wells might show upstream wells having higher cell viability (Figure 6(b3)). To investigate the DMSO control vehicle as a potential cell influencer, DMSO was deemed an actual study compound at 0.1% DMSO (i.e., 0.1 *v/v*) and dosed daily into device well 1, over 7 days (Figure 6c). At day 1, the DMSO *v/v* auto-dilutes from 0.075 (well 3) to 0.000 (well 10), with well-to-well cell viability ranges from 98% (well 3) to 97% (well 8) to 98% (well 10). At day 3, after daily re-dosing, the DMSO *v/v* auto-dilutes from 0.093 (well 3) to 0.004 (well 10), with cell viability scales 98–99%. At day 7, after daily re-dosing, the DMSO *v/v* auto-dilutes from 0.112 (well 3) to 0.047 (well 10), with cell viability scales 95–98%. As quantified, the DMSO vehicle does not influence cell culture viability across all device wells including upstream (wells 3–5), midstream (wells 6–8), or downstream (wells 9–11) culture sites. In this study, because well 11 undergoes pipette media changes, this site is considered an outlier culture well (Figure 6c well 11). In brief, the data indicates the morphological appearance of cells can be imaged with high content imaging (Cytation 5 Reader/Imager, BioTek, Winooski, VT, USA) using objective positioning software allocating for x, y, and z-height stair-step differences across the μfluidic device. Likewise, quantitative cell viability data indicate that the DMSO control vehicle can be considered a baseline-control comparison for other drug studies.

### 3.4. Use-Model, Drug Exposure, and CYP3A4 Comparisons between μFluidic Systems and Static Cultures

#### 3.4.1. DMSO Vehicle as Baseline Control for Cell Functions

We evaluated two cell culture systems, both without flow, one a conventional static culture plate and the second a static protocol in the μfluidic device (i.e., non-active μchannels). In one study, we dosed 125 μM diclofenac compound (+0.1% DMSO) on HepaRGs to evaluate CYP3A4 activity; comparatively, CYP3A4 activities were determined equivalent (data not shown). In a second study, in SsWaterfall, we dosed HepaRGs with DMSO at 24 h, 72 h, and 168 h to evaluate adjacent-well changes related to cell death, CYP3A4, and albumin; in all studies, DMSO-treated cells retained non-significant changes [23]. In subsequent cell function studies, DMSO vehicle treatments were considered as baseline control values.

#### 3.4.2. Extrapolation of Drug Exposures in μFluidic System, Diclofenac (Days 1 and 7)

Day 1: We compared CYP3A4 activities after HepaRG cells were exposed to different concentrations of diclofenac for 24 h (Figure 5 Case Study; a1–a5). The measured FITC concentration per well, column a1, is the % of FITC dispersed across the multiwell system and correlates with Figure 5a. The calculated μM diclofenac concentration, column a2, is column a1 × 125 μM dose (i.e., well 3 is 79.6% × 125 μM = 99.5 μM diclofenac). The actual μM diclofenac drug concentration, not determined, is line-shifted as FITC (389.3 g/mol) and diclofenac (296.1 g/mol) have different molecular weights, but trending flow patterns remain analogous [23]. Column a3 illustrates CYP3A4 outcomes from static culture protocols (no flow) using equivalent drug exposure concentrations. Column a4 shows CYP3A4 outcomes with cultures experiencing kinetic nutrient movements (μfluidic device). At 24 h, we did not observe any significant difference in CYP3A4 activity between the static and μfluidic systems (column a5, statistical “*p*”, ns).

Day 7: We then compared the effect of different concentrations of diclofenac on CYP3A4 activity after 7 days of exposure (Figure 5 case study; b1–b5), with daily drug re-dosing. Notably, the well-1 dose % (column b1) is listed as >1× concentration and is a function of repeat dosing with rising concentrations above the initial/starting well-1 dilutions to correlate with Figure 5b. Paraphrased, residual compound in well 1 with repeat dosing accumulates values. The % FITC concentrations are increased across all wells ranging from upstream 131.4% (well 3) to downstream 39.9% (well 11), column b1. The calculated diclofenac μM drug concentration, column b2, is column b1 × 125 μM dose (i.e., well 3 is 131.4% × 125 μM = 164.3 μM diclofenac). CYP3A4 activity in HepaRG cells was decreased in the μfluidic device wells 3–9 (column b4) as compared with the static culture (column b3), indicating a significant difference noted in column b5, statistical “*p*”. These data infer that flow in upstream well (well 3), or cascading flows across μfluidic wells 4–11, create cell-signaling concentration gradients of either dose–compound or endogenous cellular byproducts that factor into cell variances of culture responses.

### 3.5. Trending Effects on Cell Toxicity from DMSO Vehicle Control, APAP, NEF, and Indirect Cell Byproducts in μFluidic Device (Dual Toxicity Trends for Compoud Screening)

**Figure 7.**
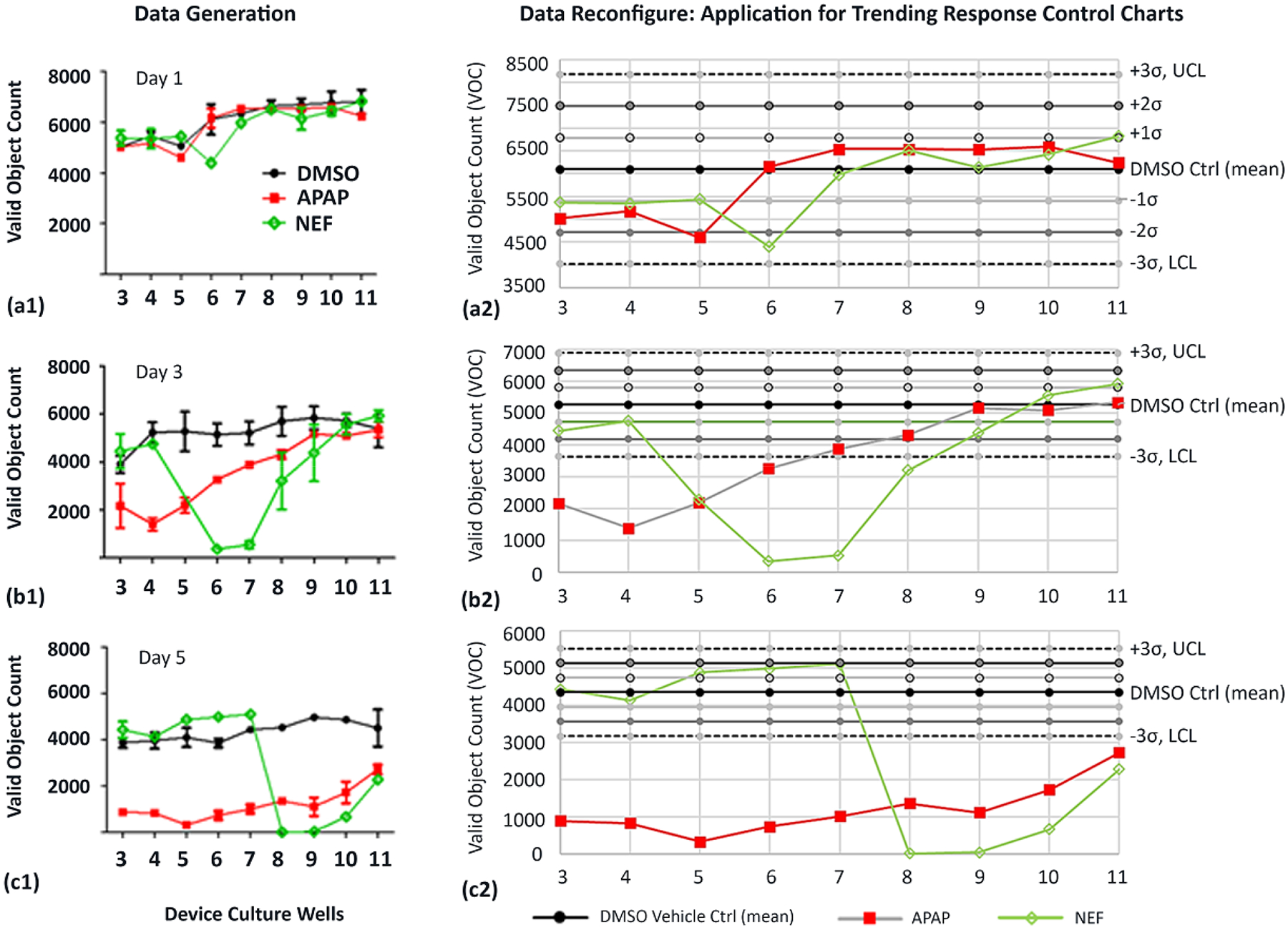
High content imaging to evaluate the variance of cell counts to indicate direct-compound or indirect-cell-byproduct toxicity locations; nuclei count (DAPI). Initially, HepaRGs were cultured for 7 days in static conditions prior to activating flow and dosing 1× daily (well 1). (**a1**,**a2**) After 1 day and a single bolus dose in well 1. The DMSO control is baseline culture while APAP and NEF are study compounds. (**b1**,**b2**) After 3 days and three bolus doses into well 1. (**c1**,**c2**) After 5 days and five bolus doses into well 1. Data are expressed as means ± SEM (n = 9). Applied statistics (**a2**,**b2**,**c2**) using six-sigma control charts with DMSO vehicle control signifying process average (mean) and ±1σ (68%), ±2σ (95%), and ±3σ (99.73%) significance offsets. If data fall outside of significant offsets, UCL or LCL, variations in the data imply special or assignable causes, (**b2**,**c2**).

High-content cell imaging was used to evaluate valid object counts (VOC: i.e., cell counts) across μfluidic platform locations, referencing device culture wells 3–11, monitoring HepaRGs at days 1, 3, and 5 (Figure 7). The four exposure compounds being DMSO vehicle control, APAP (+DMSO), NEF (+DMSO), and potential indirect exposures coming from inducible cell-generated byproducts.

#### 3.5.1. Data Generation

Day 1 signifies one day of culture after a single drug dose (Figure 7(a1)). Days 3 and 5 after three and five doses (Figure 7(b1,c1)). •At Day 1, the DMSO vehicle shows baseline VOCs (cell counts) range between 5000–6300; Figure 7(a1). Similarly, VOCs are quantified for APAP (4400–6300) and NEF (4300–6500). •At Day 3, the DMSO control has cell counts between 4000–5800; Figure 7(b1). APAP shows fewer cell numbers in upstream wells 3–5 (1200–2100), increasing cell numbers in midstream wells 6–8 (3100–4000), and equivalent DMSO control cell numbers in downstream wells 9–11 (5000–5100). NEF has DMSO equivalent numbers in upstream wells 3–4 (4400–4700), a rapid count decline in midstream wells 5, 6, and 7 (400–2000) with increasing cells numbers in downstream wells 8–11 (3100–5800). •At Day 5, the DMSO control has sustained cell counts between 3900–4100; Figure 7(c1). APAP displays much lower cell numbers across the entire channel, wells 3–11 (300–2900). NEF has DMSO equivalent numbers in upstream wells 3–7 (4100–5000) and a rapid count decline in midstream wells 8–9 (0–100), with slightly increased cells numbers in downstream wells 10–11 (800–2100).

#### 3.5.2. CV Values for VOC and Fluid-Flow Tracing across the Multiwell Channels

To illustrate intra-plate robustness of the system, coefficient of variation is used to provide a method of performance as overviewed in Table 2.

#### 3.5.3. Data Reconfigure for Trending Applications Using X Control Charts Aligned with Statistical Process Control

To expand data relevance, DMSO controls from Figure 7(a1,b1,c2) are reconfigured into lean six-sigma control charts to emphasize data-set process averages (mean) and standard deviation parameters ±1σ (68%), ±2σ (95%), ±3σ (99.73%); Figure 7(a2,b2,c2) and Table 3. Statistical boundaries for the upper control limit (UCL) and the lower control limit (LCL) are defined at DMSO ± 3σ. If study data fall outside UCL or LCL, the variation implies an observable change or unexpected nonstandard outcome (e.g., cell modulation, cell death, or generated metabolite).

### 3.6. Metabolite Generation and Dissemination in Multiwell μFluidic Culture System

#### 3.6.1. Targeting APAP Metabolites

**Figure 8.**
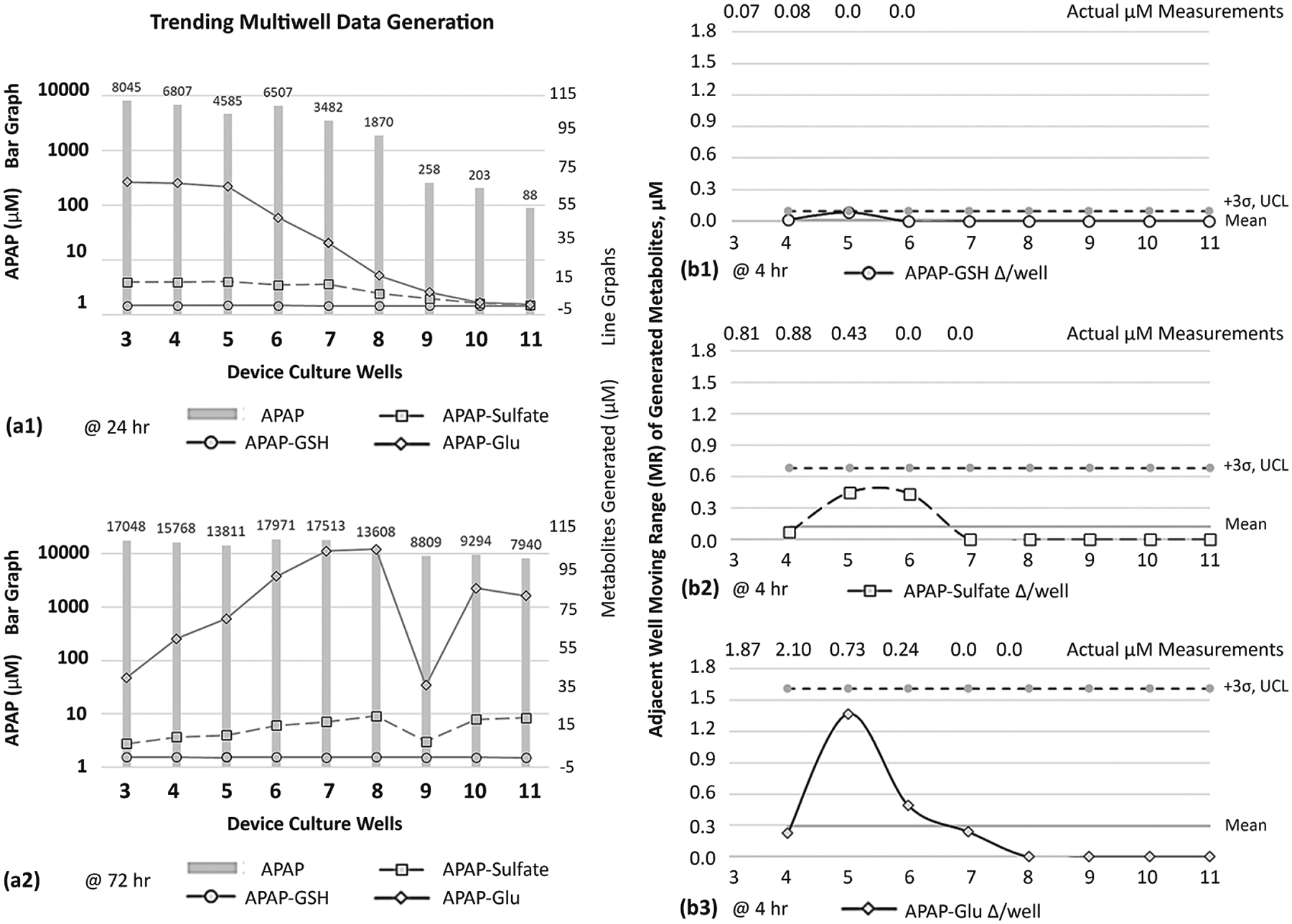
Trending channel evaluations for n = 1. (**a1**,**a2**) Concentration of APAP and cell-generated metabolites in each multiwell location, post compound dosing in the source well. *x*-axis is device culture wells 3–11, left vertical axis is APAP (logarithmic scale), right vertical axis is generated metabolites. (**a1**) After 1 day, i.e., 24 h, and a single bolus dose in well 1. (**a2**) After 3 days, i.e., 72 h, and daily dosing in well 1. (**b1**–**b3**) Origination of APAP metabolites across device multiwell locations 4–11, observed at 4 h after one APAP bolus dose into well 1. MR is the concentration change (Δ) between adjacent wells. Well 3 is devoid of an upstream adjoining well (i.e., cellular) and precludes calculation. The APAP metabolite variances (Δ/well) are (**b1**) APAP-GSH, (**b2**) APAP-sulfate, and (**b3**) APAP-Glu.

Due to the extensive literature history on APAP metabolites [45–47], APAP was targeted onto HepaRG culture wells to evaluate metabolite generation across the μfluidic channel, i.e., occurrence trends. APAP was dispensed as a bolus dose (20 mM) into well 1, once daily, and downstream media were collected from each site to measure mutable concentrations of acetaminophen and generated APAP metabolites (Figure 8). LC/MS was used to quantify concentration intensities over a timeline 0→72 h. ■At 24 h (Figure 8(a1)), gradient concentrations of APAP range from 8.045→0.088 mM (*y*-axis logarithmic scale) across the μfluidic wells 3–11 (*x*-axis). The metabolite APAP-glutathione (GSH) displays the lowest magnitude values, ranging 0.23→0.0 μM (*y*-axis line graph) across the same μfluidic wells 3–11 (*x*-axis). Metabolite APAP-sulfate has elevated magnitude values ranging 13.26→0.36 μM, while revealing activity in the last culture site, well 11. Metabolite APAP-glucuronide (APAP-Glu) has the highest magnitude values, ranging 67.7→0.8 μM, while displaying a prominent decay curve in downstream wells 6–11. ■At 72 h (Figure 8(a2)), gradient concentrations of APAP range from 17.048→7.94 mM. The metabolite APAP-GSH retains comparable 24 h displays ranging 0.21→0.0 μM. Both APAP-Sulfate 6.98→20.7 μM and APAP-Glu 39.9→103.3 μM exhibit increased levels across downstream wells 4–11, an indication of ongoing generation of metabolites. Contrastingly, well 1 reveals lower magnitudes (~0.5-fold) with the recognition that well 1 encounters highest APAP exposures. Of note, for trending appraisals, data in well 9 show a reduction in concentrations for APAP-sulfate and APAP-Glu, being possible data outliers or potential special cell-function outcomes.

#### 3.6.2. Distribution of APAP Metabolites

Given that APAP metabolites are generated in the device (Figure 8(a1,a2); line graphs), the distribution of each metabolite’s timeline, location, and shifting concentrations becomes significant exposure descriptors across μfluidic culture wells. To chronicle distribution patterns, moving-range (MR) control charts from six-sigma variability processes [41,42,48] are revamped to examine the change in biologic activity amid adjacent culture wells (Figure 8(b1–b3)). The early detection of metabolites, using LCMS, is observed 4 h after a single APAP dose into well 1, measuring for APAP-GSH (Figure 8(b1)), APAP-sulfate (Figure 8(b2)), and APAP-Glu (Figure 8(b3)). Graphically, the horizontal axis is device wells 3–11; the vertical axis is concentration Δ amid adjoining wells; the mean is the average of aggregate data points; UCL is the upper control limit calculated as 3σ. The MR activity for APAP-GSH is revealed in upstream culture wells 4 and 5, having well–well concentration Δ’s of 0.02 μM and 0.08 μM; thereafter, downstream wells 6–11 register “0” fluctuations. The MR for APAP-sulfate is displayed in upstream culture wells 4–6, with well–well Δ’s being 0.07 μM, 0.45 μM, and 0.43 μM; thereafter, downstream wells 7–11 register “0” fluctuations. The MR for APAP-Glu is displayed in upstream culture wells 4–7 with well–well Δ’s being 0.23 μM, 1.37 μM, 0.49 μM, and 0.24 μM; thereafter, downstream wells 8–11 register “0” fluctuations.

### 3.7. Metabolite Variance across Multiwell Fluidic System to Ascertain Sites of Generated Cell Byproducts

**Figure 9.**
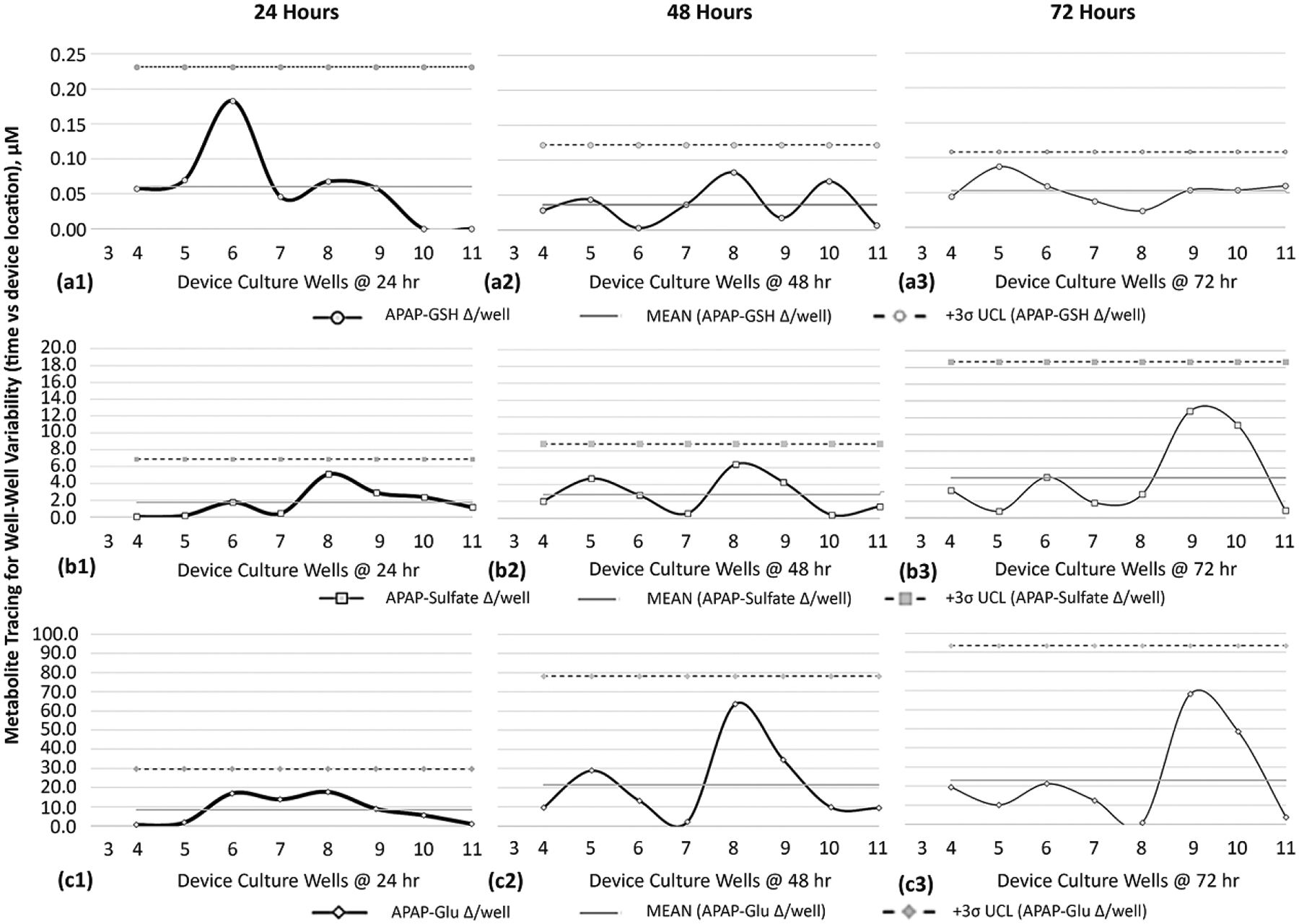
Surveillance of metabolite variability amid device multiwell locations 4–11 (n = 1), observed at 24 h, 48 h, and 72 h. MR is the concentration moving range (Δ) between adjacent wells. Well 3 is devoid of an upstream adjoining well and precludes MR calculation. Metabolites are (**a1**–**a3**) APAP-GSH. (**b1**–**b3**) APAP-sulfate. (**c1**–**c3**) APAP-Glu.

The detailed surveillance in metabolite variance, on HepaRG cells experiencing APAP exposures after 24, 48, and 72 h timelines, are presented using MR control charts to chronicle distribution patterns across device wells 3–11, Figure 9. Moving ranges are itemized by APAP-GSH-24/48/72 h (Figure 9(a1–a3)), APAP-Sulfate-24/48/72 h (Figure 9(b1–b3)), and APAP-Glu-24/48/72 h (Figure 9(c1–c3)). Graphically, accompanying descriptors include the data aggregate mean (mean) and UCL (3σ). By way of itemization, Table 4 is portioned to compare/contrast metabolite generation and dissemination. The APAP-metabolite data indicate the platform is able to allot for evaluations of cell-generated metabolites as cellular byproducts disperse across the device in compound-exposure patterns and time-distribution dynamics, allowing for trending evaluations in cell metabolite kinetics, i.e., a statistical process survey.

### 3.8. Cell Function Variance across Multiwell Fluidic System to Assess Bio-Activity Trends Affiliated with Direct Drug Exposure and Predictive Recognition for Indirect Cell-Byproduct Stimuli (Dual Assessment Screens)

Conventional cell function assays LDH, CYP3A4, and GSH are re-purposed as μfluidic device biomarkers and utilized as cell function indicators (sensors) across the platform’s changing well–well environments, i.e., monitoring worksites across an assembly line. Assays are used to track mutable cell-health kinetics, throughout μchannel wells 3–11, corresponding to real-time ramps in drug concentrations, accruing exposure times, and for gaging the incidence of cell-generated byproducts. To initiate surveys, an exposure compound is dosed into the upstream source well 1, daily for 7 days. Compounds in this study are DMSO control vehicle (0.1%), ASA (25 μM; non-DILI drug), and NEF (16 μM; DILI drug). With flow, compounds auto-disperse along the μchannel, catalyzing adaptive bioactivity amid aligned device culture site locations, i.e., wells 3–11. To reveal if gradients of drug, time, or cellular byproducts have influence on cell functions, we assessed both system-trends and adjacent-well responses, i.e., six sigma control charts, from two hepatic sources: human HepaRG cells and rat hepatocytes.

#### 3.8.1. Trending Relevance

**Figure 10.**
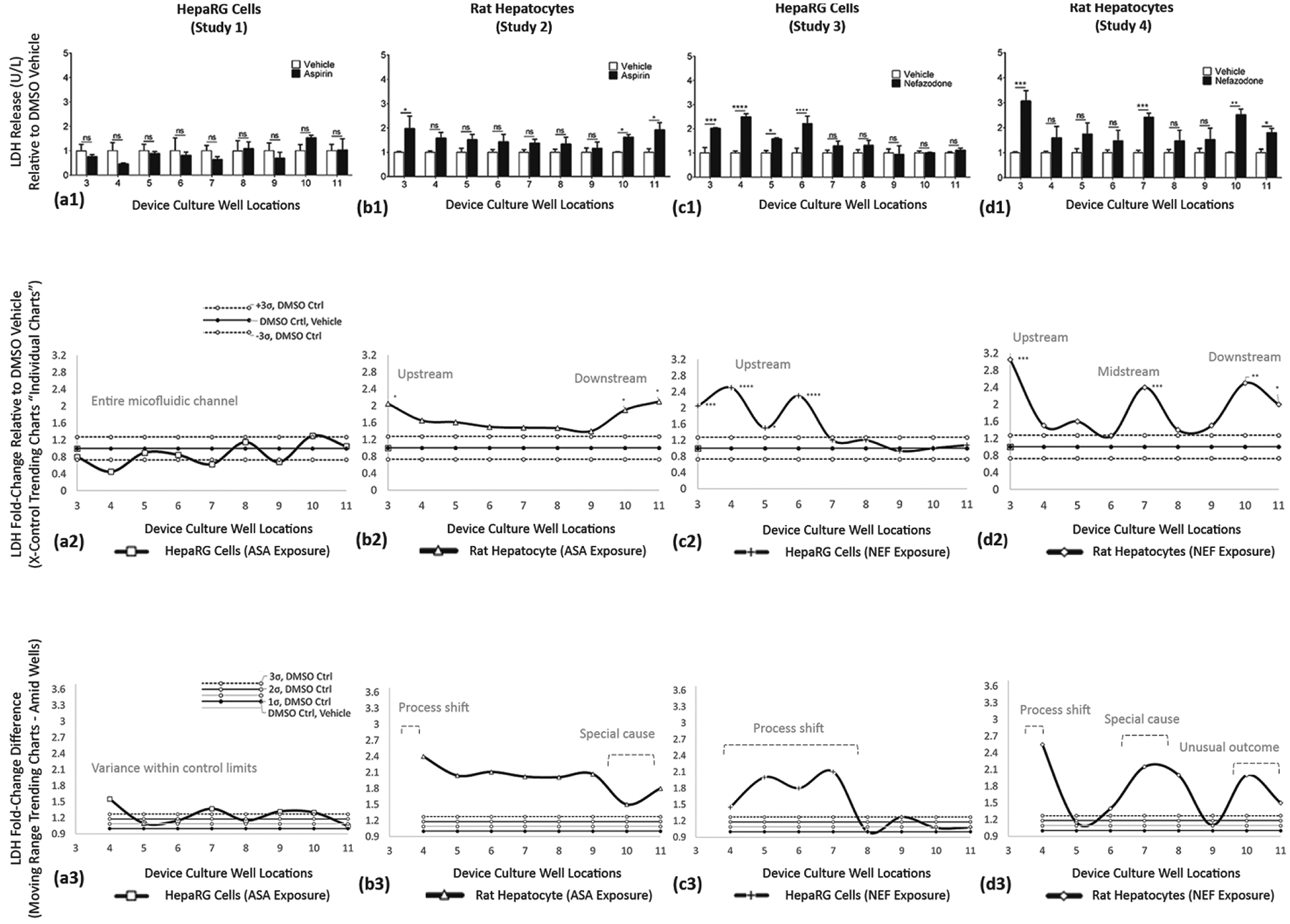
LDH release across multiwell cultures on Day 7. LDH activity shown as fold change (vertical axis) vs. DMSO vehicle controls; vehicle normalized at 1. (**a1**,**b1**) Effect of aspirin exposure in HepaRG cells and rat hepatocytes. (**c1**,**d1**) Effect of NEF exposure in HepaRG cells and rat hepatocytes. (**a2**,**b2**,**c2**,**d2**) Re-expression of data using individual trending charts (i.e., X control charts) to convey DMSO controls with drug study outcomes. (**a2—HepaRG line graph**) ASA does not induce LDH. (**b2—rat hepatocyte line graph**) ASA stimulates LDH across all wells, 3–11, with increased relevance at upstream well 3 (*) and downstream wells 10 (*) and 11 (*). (**c2—HepaRG line graph**) NEF stimulates LDH at upstream wells 3 (***), 4 (****), 5 (*), and 6 (****). (**d2—rat hepatocyte line graph**) NEF stimulates LDH across all device wells, with increased relevance at upstream well 3 (***), midstream well 7 (***), downstream wells 10 (**) and 11 (*). (**a3**,**b3**,**c3**,**d3**) Variability charts (i.e., MR control charts) to display functional fold-changes amid adjacent wells. (**a3—HepaRG line graph**) ASA does not induce a trending variation. (**b3**—**rat hepatocyte line graph**) ASA stimulates a process shift at upstream well 3 and special cause in downstream wells 10 and 11. (**c3—HepaRG line graph**) NEF induces a trending process shift at upstream wells 4–7. (**d3—rat hepatocyte line graph**) NEF stimulates a process shift at upstream well 3, a special cause at wells 7–8, and an unusual outcome in downstream wells 10 and 11. Values a1-d1 represented as the mean ± SEM (n = 9). * *p* < 0.05, ** *p* < 0.01, *** *p* < 0.001, and **** *p* < 0.0001.

**Figure 11.**
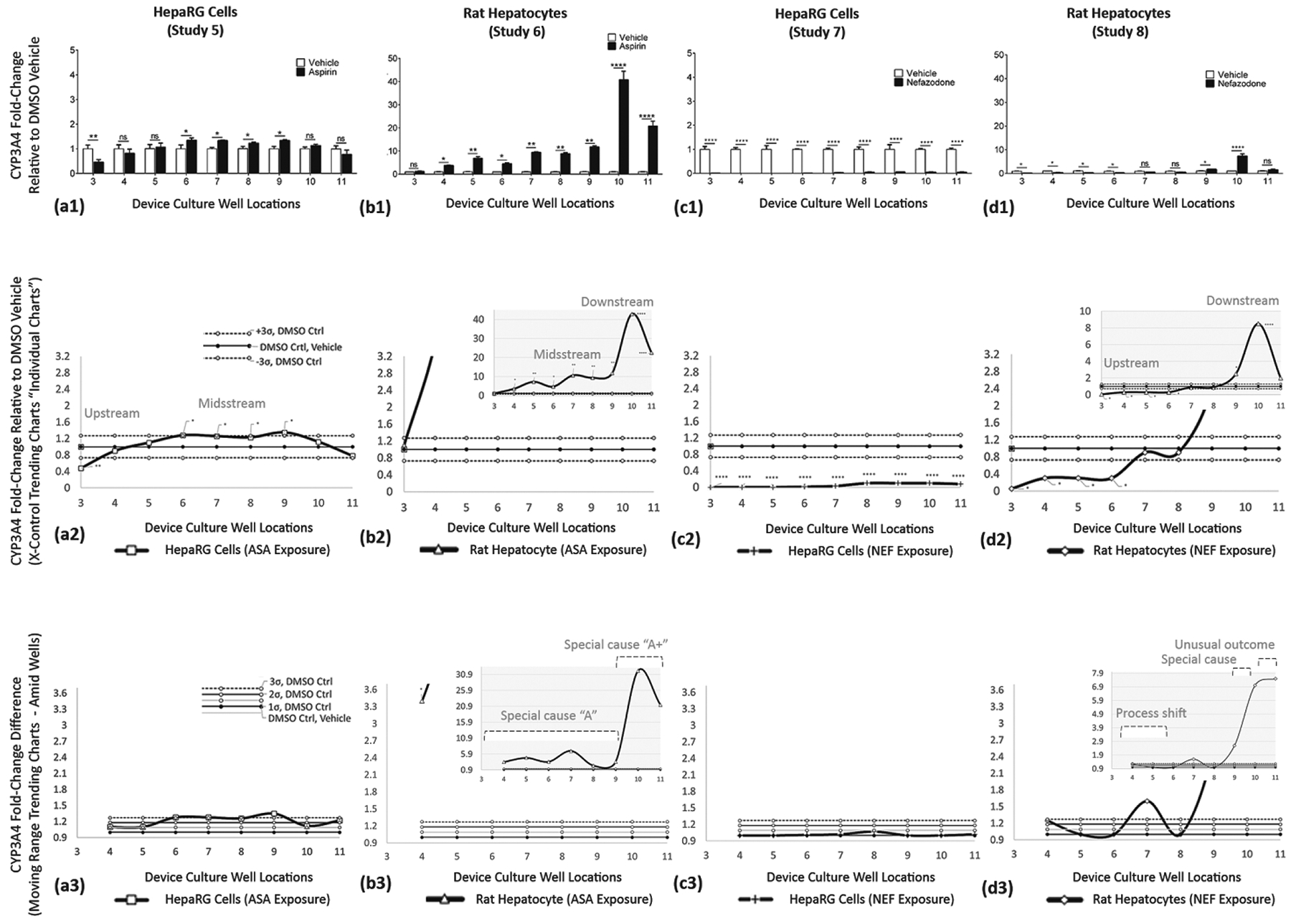
CYP3A4 enzyme activities across multiwell culture locations at Day 7. Activity shown as fold changes (vertical axis) vs. DMSO vehicle control; vehicle normalized at 1. (**a1**,**b1**) Effect of ASA exposure in HepaRG cells and rat hepatocytes. (**c1**,**d1**) Effect of NEF exposure in HepaRG cells and rat hepatocytes. (**a2**,**b2**,**c2**,**d2**) Re-expression of data using individual trending charts (i.e., X control charts) to convey DMSO controls with drug study outcomes. (**a2—HepaRG line graph**) ASA impedes CYP3A4 at upstream wells 3 (**) then shows upregulated stimulation in midstream wells 6 (*), 7 (*), 8 (*), and 9 (*). (**b2—rat hepatocyte line graph**) ASA has no effect on upstream well 3, then stimulates CYP3A4 across all remaining downstream wells 4 (*)–9 (*) and 10 (****)–11 (****). (**c2—HepaRG line graph**) NEF inhibits CYP3A4 activity across the entire μfluidic channel, inclusive of wells 3–11. (**d2—rat hepatocyte line graph**) NEF inhibits CYP3A4 activity in upstream wells 3–6 (*), regains normal function in wells 7–8, has functional increases in wells 9 (*) and 10 (****), and returns to near normal activity in downstream well 11. (**a3**,**b3**,**c3**,**d3**) Variability charts (i.e., MR control charts) to display functional fold changes amid adjacent-wells. (**a3—HepaRG line graph**) ASA induces a trending process shift of upstream inhibition (well 3) followed by midstream recovery (wells 6–9). (**b3—rat hepatocyte line graph**) ASA is inconsequential at upstream well 3, stimulates a special cause “A” in wells 4–9, then an upregulation special cause (A+) in downstream wells 10 and 11. (**c3—HepaRG line graph**) NEF induces a negative trending process shift across the entire μchannel. (**d3—rat hepatocyte line graph**) NEF creates a negative process shift, wells 3–6, normal functions wells 7–8, special cause wells 9–10, and unusual decline outcome in well 11. Values a1-d1 are represented as the mean ± SEM (n = 9). * *p* < 0.05, ** *p* < 0.01, and **** *p* < 0.0001.

**Figure 12.**
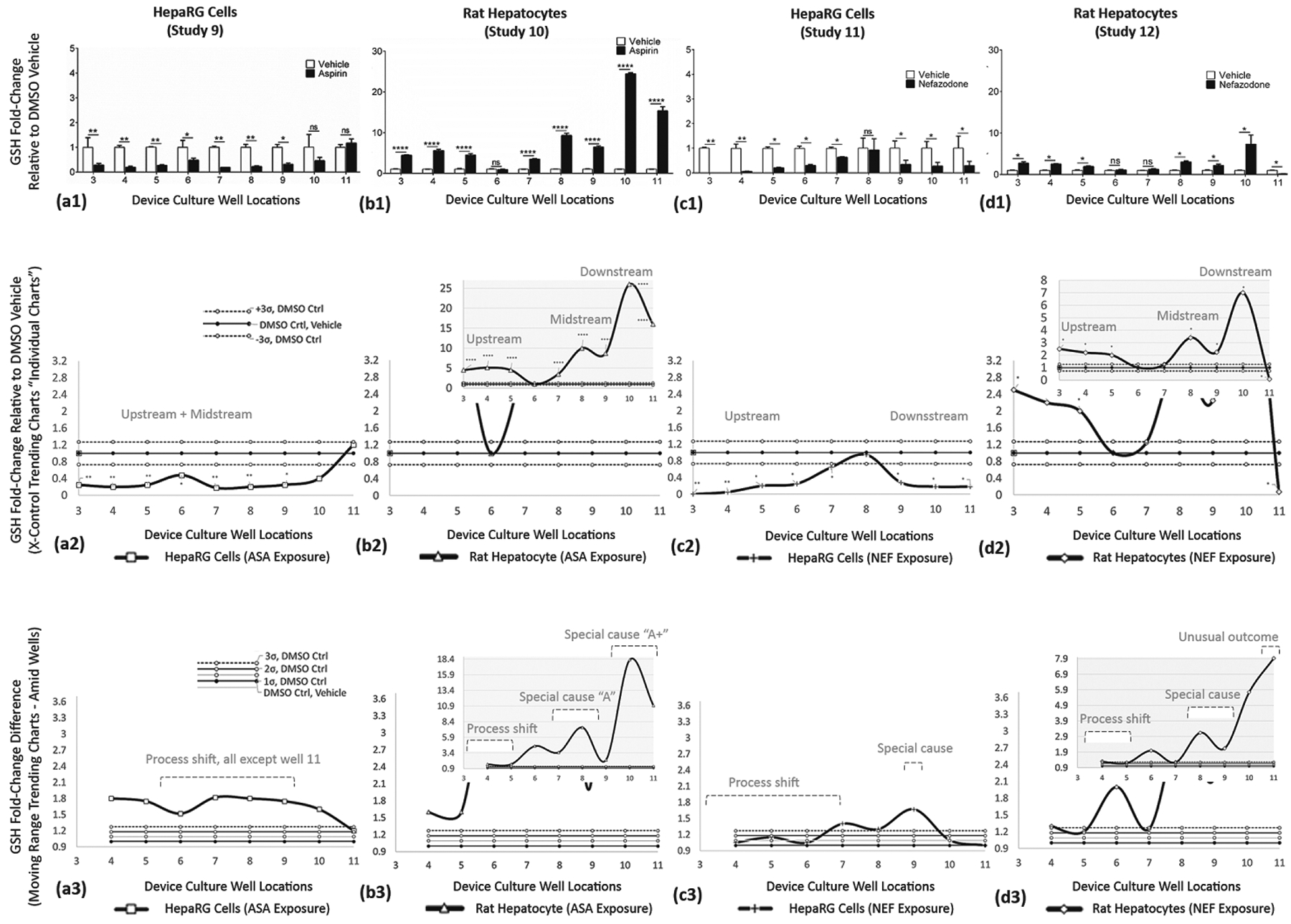
GSH levels across multiwell culture locations at Day 7. GSH synthesis shown as fold changes (vertical axis) vs. DMSO vehicle control; vehicle normalized at 1. (**a1**,**b1**) Effect of aspirin exposure in HepaRG cells and rat hepatocytes. (**c1**,**d1**) Effect of nefazodone exposure in HepaRG cells and rat hepatocytes. (**a2**,**b2**,**c2**,**d2**) Re-expression of data using individual trending charts (i.e., X control charts) to convey DMSO controls with drug study outcomes. (**a2—HepaRG line graph**) ASA inhibits GSH activity across wells 3 (**)–9 (*). (**b2—rat hepatocyte line graph**) ASA promotes GSH synthesis in upstream wells 3 (****)–5 (****), displays normal function in well 6, then stimulates GSH functions in wells 7 (****)–11 (****). (**c2—HepaRG line graph**) NEF inhibits GSH activity across wells 3 (*)–7 (*) and wells 9 (*)–11 (*). (**d2—rat hepatocyte line graph**) NEF promotes GSH synthesis in upstream wells 3 (*)–5 (*), displays normal function in wells 6–7, again stimulates GSH functions in wells 7 (*)–10 (*). (**a3**,**b3**,**c3**,**d3**). Variability charts (i.e., MR control charts) to display functional fold changes amid adjacent wells. (**a3—HepaRG line graph**) ASA induces a trending process shift across most of the μchannel, wells 3–10. (**b3—rat Hepatocyte Line Graph**) ASA stimulates a process shift in wells 3–5, has normal function at well 6, stimulates a special cause “A” in wells 7–9, then enhanced special cause “A+” in wells 10–11. (**c3—HepaRG line graph**) NEF induces a trending process shift in wells 3–7, normal function at well 8, then a negative special cause in wells 9–11. (**d3—rat hepatocyte line graph**) NEF stimulates a process shift in wells 3–5, has normal function in wells 6–7, stimulates a special cause in wells 8–10, then unusual outcome in well 11. Values (**a1**–**d1**) are represented as the mean ± SEM (n = 9). * *p* < 0.05, ** *p* < 0.01, and **** *p* < 0.0001.

**Figure A1.**
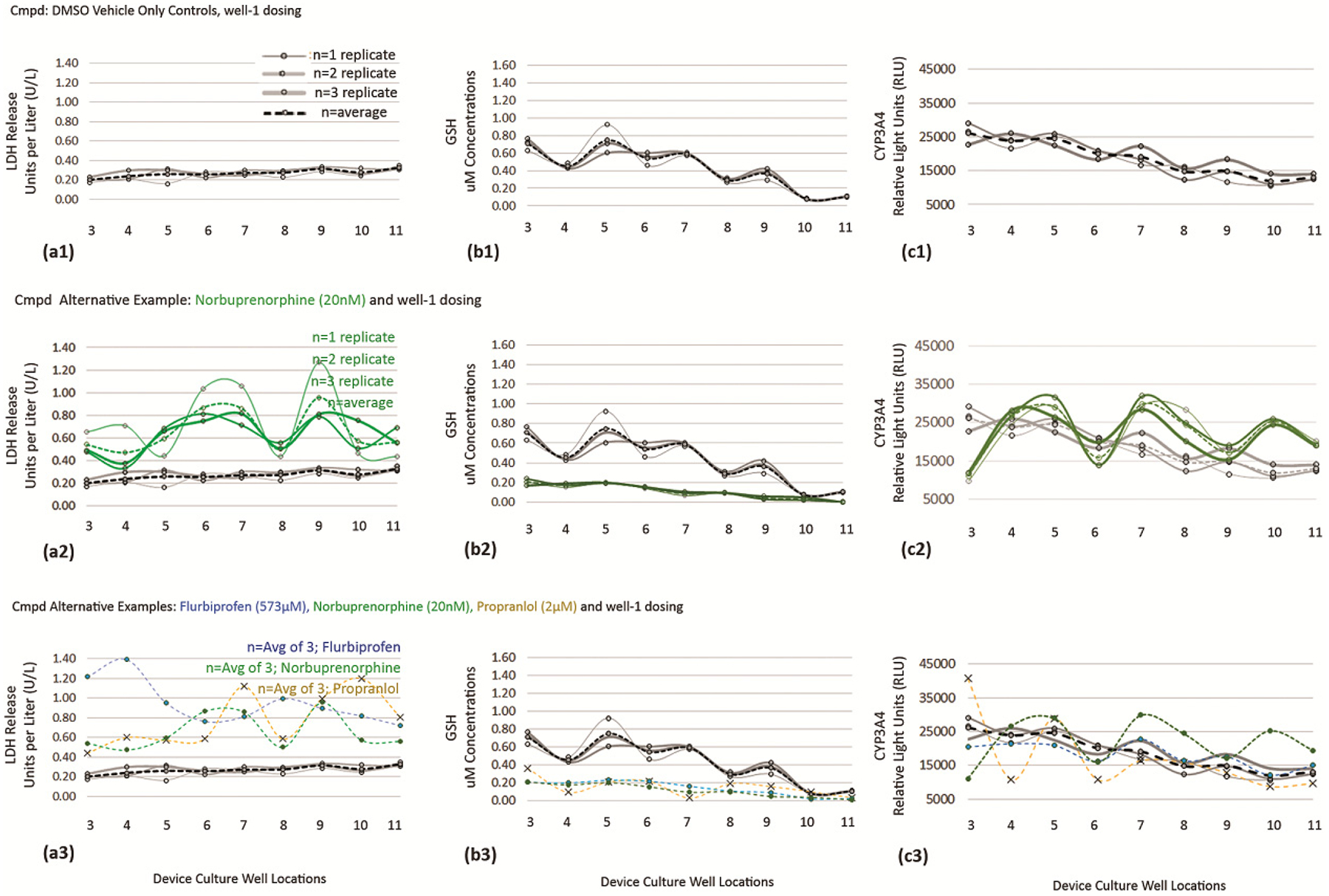
DMSO controls and drug data replicates. (**a1**,**b1**,**c1**) DMSO controls only. (**a2**,**b2**,**c2**) DMSO controls vs. compound norbuprenorphine. (**a3**,**b3**,**c3**) DMSO controls vs. compounds flurbiprofen, norbuprenorphine, and propranlol.

Study outcomes of discrete, continuous, and trending models are displayed in Figures 10–12; LDH (Figure 10), CYP3A4 (Figure 11), and GSH (Figure 12). In figures, vertical columns are categorized (a1–a3) HepaRGs exposed to ASA, (b1–b3) rat hepatocytes exposed to ASA, (c1–c3) HepaRGs exposed to NEF, and (d1–d3) rat hepatocytes exposed to NEF. Within figures, horizontal rows are demarcated by a1→d1, a2→d2, and a3→d3 as defined in Table 5. From the same SOP, trending effects of DMSO vehicle, additional exposure compounds, and additional assays are corroborated in Appendix A with Figure A1 and in the supplemental section as illustrated in supplemental Figure S1.

##### LDH ASSAY—Functional Indicator (Studies 1–4)

LDH is found in cells. LDH discharge is used as a biomarker of cell cytotoxicity as release can increase mitochondrial damage [19]. **ASA exposure on HepaRG cells (Study 1)** is displayed in Figure 10(a1). LDH outcomes, for DMSO vs. ASA, are non-significant (ns) as revealed in wells 3–11. **ASA exposure on rat hepatocytes (Study 2)** is displayed in Figure 10(b1). ASA induces LDH release in upstream well 3 (*) and downstream wells 10 (*) and 11 (*). **NEF exposure on HepaRG cells (Study 3)** is displayed in Figure 10(c1). NEF stimulates LDH in upstream wells 3 (***), 4 (****), 5 (*), and 6 (****); thereafter, wells 7–11 register as non-significant cell function activities. **NEF exposure on rat hepatocytes (Study 4)** is displayed in Figure 10(d1). NEF stimulates LDH in upstream well 3 (***), midstream well 7 (***), and downstream wells 10 (**) and 11 (*). Interior wells 4–6 and 8–9 register as non-significant. For Studies 1–4, LDH trending responses are further summarized in Table 6.

##### CYP3A4 ASSAY—Functional Indicator (Studies 5–8)

Cytochrome P450 3A4 activity is found in the liver and has a significant role in the biotransformation of compounds [49]. **ASA exposure on HepaRG cells (Study 5)** is displayed in Figure 11(a1). ASA impedes CYP3A4 activity in well 3 (**) with slightly upregulated activities in wells 6 (*), 7 (*), 8 (*), and 9 (*); remaining wells 4, 5, 10, and 11 register as non-significant. **ASA exposure in rat hepatocytes (Study 6)** is displayed in Figure 11(b1). In data presentation, the scale of the vertical axis increased 10-fold to accommodate for intensified CYP3A4 outputs, i.e., range 0–5 adjusted to 0–50. ASA exposure is non-significant in well 3, CYP3A4 upregulates in wells 4 (*)–9 (**) to denote 3-to-10-fold increases over DMSO controls, then maximized in wells 10 (****) and 11 (****) at 40-fold and 20-fold levels. **NEF exposure in HepaRG cells (Study 7)** is displayed in Figure 11(c1). NEF exposure shows no CYP3A4 activity in wells 3–6 and marginal 0.1fold functions in wells 7–11. Throughout, DMSO vehicle controls have higher CYP3A4 over NEF datasets, wells 3 (****)–11 (****). **NEF exposure in rat hepatocytes (Study 8)** is displayed in Figure 11(d1). In data generation, the scale of the vertical axis is increased 10-fold to accommodate intensified CYP3A4 outputs, i.e., range 0–5 adjusted 0–50. The NEF datasets show impeded CYP3A4, below DMSO vehicle controls in wells 3 (*)–6 (*), data being non-significant in wells 7–8, then NEF has higher CYP3A4 activity in wells 9 (*)–10 (****), concluding with non-significant cell reactions in well 11. For Studies 5–8, CYP3A4 trending responses are further summarized in Table 7.

##### GLUTATHIONE (GSH) ASSAY—Functional Indicator (Studies 9–12)

Glutathione (GSH), a tripeptide present in most tissues, is highly concentrated in the liver [50]. GSH protects against oxidative stress and regulates important events such as growth and apoptosis. **ASA exposure in HepaRG cells (Study 9)** is displayed in Figure 12(a1). ASA impedes GSH activity in wells 3 (**), 4 (**), 5 (**), 6 (*), 7 (**), 8 (**), and 9 (*); wells 10–11 register as non-significant. **ASA exposure in rat hepatocytes (Study 10)** is displayed in Figure 12(b1). The scale of the vertical axis is increased 6-fold to accommodate for intensified GSH outputs, i.e., range 0–5 adjusted 0–30. ASA stimulates GSH response in wells 3 (****)–5 (****) with activity approaching 5.8-fold, then non-significant at well 6, then inducible again in wells 7 (****)–11(****) with activity 26.5-fold above DMSO controls. **NEF exposure in HepaRG cells (Study 11)** is displayed in Figure 12(c1). NEF impedes GSH activity in wells 3 (**), 4 (**), 5 (*), 6 (*), and 7 (*) having 0 to 0.25-fold ranges, is non-significant in well 8, then impedes activity in wells 9 (*), 10 (*), and 11 (*), having 0.2 to 0.3-fold ranges. **NEF exposure in rat hepatocytes (Study 12)** is displayed in Figure 12(d1). The scale of the vertical axis is increased 6-fold to accommodate for intensified GSH outputs, i.e., range 0–5 adjusted 0–30. NEF stimulates GSH activity in wells 3 (*), 4 (*), 5 and (*), is non-significant in wells 6–7, induces again in wells 8 (*), 9 (*), and 10 (*), then falls below DMSO control in well 11 (*). For Studies 9–12, GSH trending responses are summarized in Table 8.

#### 3.8.2. Vetting Trends in Cell Modulation by Drug or Cell Byproduct Stimuli (Studies 1–12)

LDH, CYP3A4, and GSH cell indicator data are coalesced to ascertain if exposure compound or endogenous cell byproducts induce cell function variance across device worksites (Table 9). In Table 9, the twelve aforementioned studies are itemized with odd study numbers being HepaRGs (1, 3, 5, 7, 9, and 11) and even study numbers featuring rat hepatocytes (2, 4, 6, 8, 10, and 12). The table’s vertical columns are cataloged into cell phenotype, exposure drug, cell function, and device culture well sites for upstream (wells 3–5), midstream (wells 6–8), and downstream (wells 9–11) workspaces. Subcategorized are statistical classifications using six-sigma nomenclature, for cell function activities, to indicate a process shift (direct drug influence; light grey backdrop), w/i normal limits (not significant; white backdrop), and special cause or unusual outcome (cell byproduct/metabolite influence; dark grey backdrop). As demarcated, all **HepaRG** studies initiate with a process shift, i.e., direct drug influence, except Study 1. Study 1 is the LDH response from aspirin exposure (HepaRG/ASA/LDH; Figure 10(a1–a3)) displaying no significant change when compared against the baseline DMSO control (i.e., w/i normal limits). Comparatively, distinct studies show cell functions remain under direct drug influence (HepaRG/NEF/CYP3A4; Figure 11(d1–d3)) while other studies rebound to achieve normal cell functions aligned with baseline DMSO controls (HepaRG/ASA/GSH; Figure 12(a1–a3)). Probingly, Study 5 (HepaRG/ASA/CYP3A4; Figure 11(a1–a3)) and Study 11 (HepaRG/NEF/GSH; Figure 12(c1–c3)) allude to midstream normal functions, i.e., w/i control limits, with reduced downstream cell activities to indicate marginal levels of special cause effects (i.e., cell byproducts). In comparison, assessing **rat hepatocytes**, all studies initiate with a process shift, i.e., direct drug influence, except Study 6. Study 6 is the CYP3A4 response from aspirin exposure that remains within DMSO baseline controls (rat Hep/ASA/CYP3A4; Figure 11(b1–b3)). For other companion studies (Table 9; Studies 2, 4, 8, 10, 12), cell activities rebound into normal cell function levels, i.e., w/i control limits. Thereafter, all downstream cell functions modulate again to indicate a special cause (i.e., cell byproduct) or unusual or tertiary outcome (i.e., 2nd cell byproduct), inclusive of LDH, CYP3A4, and GSH compound appraisals. Studies having both special and tertiary variances are Study 4 (Rat Hep/LDH/NEF; Figure 10(d1–d3)) and Study 12 (rat Hep/GSH/NEF; Figure 12(d1–d3)). By proportion, HepaRG studies experience 33.3% special cause outcomes, albeit displaying minimal effects, while rat hepatocyte studies experienced 100% special cause outcomes and reveal significantly high magnitude differences for CYP3A4 (Figure 11b,d) and GSH (Figure 12b,d), 7.9-fold to 40-fold, respectively. The high-magnitude responses inferring rat hepatocytes have elevated sensitivity outputs related to cell indicator functions. In brief, the variance of cell functions across device culture wells can reveal if an exposure compound has no influence (i.e., w/i normal limits), has direct drug influence (i.e., process shift), or has indirect drug influence (i.e., special cause from cell byproducts/metabolites). In this manner, when variance trends are detected (i.e., special cause or tertiary), follow-up studies using LC/MS (discovery mode) could be resourcefully applied, via compound targeting, so previously unspecified cell byproducts are identified into metabolite inducers. That is, efficiently, logically, and resourcefully finding the unknowns.

## 4. Discussion

### 4.1. Drug Development Is a Very Long and Expensive Process

A pharmaceutical compound can take 10–15 years to progress through the development process before FDA clearance. Depending on the type of drug being developed, the total cost per compound can run USD 1.8B–5.0B [51–54]. The process is plagued by late-stage clinical failures, which could be circumvented to some degree by implementing innovative tissue engineering *in vitro* testing earlier in the development pipeline. A goal being “fail fast” in early research stages to avoid expended time, squandered resources, and depleted finances. Many companies attempt to improve efficiency in drug development by employing a battery of front-end *in vitro* assays, these being efforts to distinguish lead compounds for toxicity and efficacy. Still, the evolution of predictive science continues to result in false-negative (missing potentially unsafe compounds) and false-positive (falsely identifying potentially efficacious compounds as toxic) outcomes. Similarly, the use of *in vivo* animal models has become more questionable as they often fail to identify safety liabilities that ultimately arise in human trials. One *in vivo* study, comparing drug toxicities between human and animals, indicated that only 43% of animal models can predict human toxicity [55]. Correspondingly, many political and societal pressures are driving the effort to reduce or eliminate the use of animal studies in toxicological research [56,57]. Overall, a critical unmet need persists for innovative *in vitro* physiologically tools that can improve predictive potentials for evaluation of new drug candidates.

### 4.2. Natural 3D Organs with Instrinsic Fluid Flows

Native organ systems, human or animal, innately have *in vivo* fluid movements from velocity and diffusion, i.e., dispersion, together being flow vigors to help facilitate exposures of compounds, nutrients, and metabolites [58]. In contradiction, *in vitro* drug research historically employs static cultures for evaluating compounds [59]. These no-flow schemes are absent of delivery velocity, are limited in transport diffusion, and lack dynamic exposure conditions intrinsic to 3D natural organ systems. As such, traditional “no-flow” classifications are disadvantaged in schemes to predict efficacy and toxicity of compounds, are inefficient in monitoring gradient ramps of drug concentrations (e.g., nonlinearity and redosing), and are perceived incomplete for evaluating cell byproducts for metabolite interfacing. Accordingly, contemporary tissue engineering processes are impelled to provide *in vitro*, multifarious, and complex models having rapid, accurate, and focused predictions for compound evaluations [60–62].

#### 4.2.1. μFluidic Contemporary Technologies for In Vitro Cell Simulations

Already, rival μfluidic culture systems provide use-model benefits having dissimilar microenvironments with discrete applications and distinct advantages [63,64]. For evolving technologies, Kirkstall’s Quasi Vivo allows Lego-like building systems. Hurel’s Hurelflow allows co-cultures and reactive metabolites. BellBrook’s IUVO allows cell invasion analysis. BioIVT’s HepatoPac allows cell-patterned modules. Various commercial systems having fit-for-purpose functions include Millipore’s CellAsic with flow gradients, CNBio having transwells and basal flow, Emulate with microchannel scaffolds, TissUse with systemic organics, Mimetas with vascular Phaseguides, and inSphero with 3D hepatic co-cultures. A synopsis of simulations includes drug screening, cell signaling, proliferation, and differentiation, as overviewed in Table 10.

#### 4.2.2. Unmasking the Cell-Byproduct Mechanism Using 1-Way In Vitro μFluidics

Static systems have limits in identifying drug toxicity due to a lack of separation between parent-compound and cell-metabolite, i.e., masking cell-byproduct mechanisms. Depending upon the rate of metabolism, toxicity may be missed in a static system or a drug’s safety may be grossly overestimated. In static systems, slowly metabolized compounds appear to be much safer because the standard protocol involves complete media changeover every 24–48 h. Total media changes remove all cell byproducts from the static system and replenish the parent compound; thus, creating a need to dose at exceedingly high parent compound concentrations to accumulate enough metabolites to detect any toxicity—a limitation of short incubation times provided in static techniques.

Recirculating μfluidic systems have limits in identifying metabolite toxicity as cell secretions are blended into the common nutrient media and this mixture is repeatedly transmitted back onto the same culture spaces, i.e., masking individual cell-byproduct mechanisms.

Unidirectional μfluidic systems, i.e., 1-way flows, are able to preserve cell byproducts to allow for enhanced detection of metabolite-mediated toxicity of slowly metabolized compounds as conserved cell secretions are used to evaluate de-risking of toxicity profiles by way of concurrent mechanistic evaluations of both ascending primary compound (i.e., drug) and perpetually generating secondary metabolite exposures (i.e., cell byproducts).

#### 4.2.3. Trending Variations to Observe Different Types of Cell Toxicity

The SsWaterfall platform is a screening tool with the potential to evaluate wide-ranging compound exposures and concurrently observe indirect cell byproduct influencing amid stable, upregulated, or downregulated cell functions. The outcomes are non-specific by design, such that all-inclusive datasets are utilized as additional information categories (i.e., cell responses previously unviable) to be co-analyzed with traditional culture techniques. From an analogous viewpoint, nuclear magnetic resonance (NMR) metabolite snapshots are all-inclusive, non-specific, and powerful datasets that provide generalized bulk information (e.g., inclusive cell byproducts), whereas liquid chromotography mass spectrometry (LCMS) is specific to one deliverable such as one evaluated metabolite. In this NMR–LCMS scenario, NMR can be utilized to evaluate all-inclusivity that affords information to guide effective and efficient LCMS studies; that is, bulk analysis as resourceful tailoring into specific details. In this regard, the SsWaterfall platform’s aim is to recognize unforeseen cell-modulation trends early in research and development *in vitro* stages, as generalized non-specific data snapshots. For a study, if data trends of primary compound and indirect cell byproducts remain stable, e.g., do not modulate, the implication is that the compound is not detrimental to cell health. However, if culture modulations are recognized, then additional and precise evaluations of the compound are needed as drug developers better understand unanticipated incidences to include inducible cell byproducts. Herein, drug developers may quickly weed-out the compound to avoid lost time, lost effort, and lost money corresponding to the fail-fast mantra.

### 4.3. DILI Is One Reason That New Compounds Are Removed from Late-Stage Clinical Trials

In the framework of precision medicine, DILI is a subgroup of liver injury and is one reason that new drug compounds are removed from late-stage clinical trials and after market entry [65–67]. Typically, static cultures or animal studies are deemed unreliable because DILI can be induced by parental drugs or subsequent cell-generated metabolites [55], indicating DILI as a longer-term chronic hepatotoxicity injury model with explicit cell signaling implications, whereas short-term *in vitro* cultures of hepatocytes, in static setups, are limited due to omitted cell signaling dynamics. Meanwhile, longer-term *in vivo* animal studies can be limited by species-specific cell metabolite generation of contradictory complexities. In consequence, *in vitro* methods to predict DILI with high accuracy continue to be sought-after attributes. Suitably, fluid-flow systems are evolving to replicate, as accurately as practical, *in vivo* exposure schemes. Given that hepatocytes are the performing biologic participants, and distinct cell phenotypes have unique functional responses, both the adequately studied human HepaRG cell line [37,68] and historically analyzed primary rat hepatocytes are considered stable reproducible contributors needed for these exposure investigations. Relatedly, isolated primary human hepatocytes were excluded from initial studies due to their inherent cell function variability [69], but primary human cells could be available for accommodating evaluations.

### 4.4. Perpetually Adapating Cell Responses for Complementary Knowledge

With flow, the operational use of hepatocytes in culture facilitates extended culture timelines, retains cell function efficacy, and maintains activities of metabolizing enzymes [70]. Collectively, by emulating a radiation AUC exposure model, the device supports atypical cell responses to create complementary data outcomes, i.e., not replicate or replacement data. Each μchannel workspace experiences quantifiable-centric contacts, compound-centric exposures, and accumulation-centric encounters (Figure 4(b1–b3)). Herein, the multiwell μfluidics device is designated a biologic process assembly line for both compound-exposure and cell-generated byproducts, where real-time trending variations are used to evaluate bio-transformations. Analogous trending charts are successfully employed in conventional manufacture assembly-line processes, i.e., six-sigma engineering analysis, to track, sustain, or re-align production processes within boundary limits (e.g., UCL and LCL).

#### 4.4.1. Focused Modeling with Enhanced Tissue Engineering

Traditionally, in efforts to predict the bioactivity responses of new compounds, drug development companies have devoted enormous *in vitro* research efforts for predictive efficacy and foretelling toxicity, by employing high-throughput cell culture systems. Still, the abundance of front-end testings utilized, i.e., extensively searching for validations, continues to generate findings that do not fully recapitulate *in vivo* effects and results in late-stage clinical failures at significantly high costs. Many shortfalls could be overcome through enhanced tissue engineered models that more robustly mimic natural *in vivo* organ structures. Focused modeling could have explicit and tangible deliverables contrary to the voluminous routine of implicit studies, i.e., evaluating subgroups in high accuracy formats. By evolving complex organ structures, *in vitro* assemblies might exist as viable and non-viable influencers to include nutrient mass transport, cell communication, and cell generated byproducts. In this framework, multiwell and μfluidic platforms are expected to enrich supplemental datasets to help select/deselect compounds of interest.

#### 4.4.2. LDH Release Is a Biomarker to Evaluate Liver Damage

LDH release in the blood is an indicator of cell death and disintegration of the cell membrane. ■LDH release from HepaRGs was non-significant from ASA exposure (Figure 10a). LDH release from HepaRGs had upstream significance from NEF exposures (Figure 10c). ■LDH release from rat hepatocytes was more sensitive with up/downstream significances from ASA exposure (Figure 10b) and up/mid/downstream significances from NEF exposure (Figure 10d). United, these findings suggest that trending variations in the μfluidic system can be used to predict compound influence via modulations in cell functions or differentiating cell health (i.e., upstream process shifts to include toxic influences) and concurrently evaluate indirect bio-transformations from their sequential downstream cell-generated byproducts (i.e., consequent special cause and unusual outcomes). This *in vitro* screening outcome generates general cell-byproduct information, non-specific by design, to signify weed-out selections or to indicate that additional compound evaluations may be necessary before in-vivo studies.

#### 4.4.3. CYP3A4 Is the Most Critical Drug-Metabolizing Enzyme Expressed in Liver Cells

CYP3A4 is involved in phase I metabolism of xenobiotics and participates in the metabolism of many clinical drugs [71]. ■CYP3A4 also contributes to remarkable first-pass elimination of its substrates, modulations being essential in this study. ■CYP3A4 activity from rat hepatocytes exposed to ASA showed as non-significant upstream, upregulated midstream, and modified again downstream (Figure 11b). Comparable high-sensitivity responses are derived from rat hepatocytes exposed to NEF (Figure 11d). ■CYP3A4 bioactivity from HepaRG cells was decreased with high-dose ASA at upstream sites, then nominally upregulated during lower-dose midstream exposures (Figure 11a); findings consistent with the literature reports that ASA increases CYP3A4 activity [72]. CYP3A4 bioactivity from HepaRG cells was decreased by NEF showing negligible activity, far below DMSO controls (Figure 11c); findings consistent with the literature reports that NEF is a substrate and inhibitor of CYP3A4 *in vitro* [73] and an inhibitor of CYP3A4 *in vivo* [74]. United, these findings suggest that cell-generated byproducts are low in HepaRGs and high in rat hepatocytes, having inducible trends of CYP3A4 activity.

#### 4.4.4. Glutathione Is Produced in All Mammalian Cells

Glutathione has secreted forms of thiol-reduced (GSH) and disulfide-oxidized (GSSG). GSH is the primary form, 90% is in the cytosol and 10% in the mitochondria and endoplasmic reticulum (ER). GSH is involved in several vital functions including (i) scavenging free radicals; (ii) detoxifying electrophiles; (iii) providing a reservoir for cysteine; (iv) maintaining the essential thiol status of proteins; and (v) modulating critical cellular processes such as growth and death, immune function, and fibrogenesis [75]. The liver has the highest GSH level and plays a crucial role in interorgan GSH homeostasis. Reduced hepatic GSH levels can exacerbate and perpetuate liver injury, modulations being essential in this study. ■HepaRG cells exposed to ASA or NEF decreased GSH across most worksites (Figure 12a,c), suggesting exposures induce a reduction in expression of the genes involved in GSH synthesis. ■Opposingly, rat hepatocytes exposed to ASA or NEF had significantly higher GSH for up/mid/downstream μfluidic sites (Figure 12b,d), possibly inducing hepatocyte proliferation [76,77]. United, these results suggest predictable dysregulation of GSH synthesis after treatment.

#### 4.4.5. Observing Cell Behaviors

Historically, APAP is a well-studied drug standard having known metabolites APAP-glucuronide (APAP-Glu), APAP-sulfate, and APAP-glutathione (APAP-GSH) [45–47]. The targeting of APAP metabolites within the μfluidic device, initially, confirmed that multiwell behaviors are consistent with known APAP compound activities as three metabolites did generate across the μfluidic channel, Figures 8 and 9. Consequently, new information includes APAP concentrations versus metabolite generation levels, accruing timelines of APAP exposures related to up-/mid−/−downstream sites of metabolite formation, and revealing how signals from adjacent wells may influence cyclical pattens in metabolite behaviors (Figure 9). In Figure 9, APAP-GSH displays the highest cell function variability at 24 h (well 6), whereas APAP-sulfate and APAP-Glu display most function variability at 72 h in downstream culture wells 9–10.

Previously, in 2003, Nefazodone was removed from the market after 9 years as an antidepressant product (Serzone) [78]. Discontinuation was due to adverse hepatic events and hepatic injury as listed in the World Health Organization database of adverse drug reactions, with severe reactions involving elevated bilirubin and onset of jaundice, hepatitis, and hepatocellular necrosis. With respect to time intervals, cases of hepatotoxicity usually occurred within the first 4–8 months of starting the drug. Still, the mechanism with which nefazodone causes injury remains unknown, with scientific relevance focused on P450-CYP3A4, as nefazodone is both metabolized and is an inhibitor of this enzyme. Dual functionality, induction/inhibition, indicates that competing P450-CYP3A4 drugs could delay or increase nefazodone clearance. Herein, hepatotoxicity may be mediated by toxic intermediates of its metabolism as cell byproducts influence downstream hepatic structures initiated from hepatic zone 1 (periportal), progressing through zone 2 (intermediary), and influencing zone 3 (perivenous) locals. In patient cases of acute hepatitis, liver biopsy usually demonstrated variable degrees of centrolobular zone-3 necrosis [79]. By trending comparisons, the μfluidic cell responses shown in Figure 7(b1–c1) display downstream cell death, an influence of cell byproducts, as upstream culture sites continue to remain viable.

In μfluidic devices, 1-way flow creates ascending compound exposures that are different from well 3 to well 11, Figure 4(b1). Gradient ramps may allow cell cultures to craft resistance against compounds. In auxiliary assessments (data not shown), select cell studies revealed two unique corollaries amid fluidic vs. static cultures: (i) cell function responses from μfluidic cultures were delayed by 3–5 days prior to achieving similar outcomes as derived from static cultures, and (ii) a resistance response from μfluidic cultures allowed cells to tolerate 2×–3× higher compound concentrations prior to harmful responses, as quantified from static cultures. Herein, the ramp of compounds may have relevance to *in vivo* studies as the natural body ramps exposures by repeat dosing.

### 4.5. Emerging Advantages with Continued Optimization

The *in vitro* μfluidic system can help evaluate trending variances of drug–drug interactions between compounds and their cell generated byproducts, with importance of fluid-flow for liver culture models [58]. Healthy hepatocytes require a relatively massive influx of oxygen difficult to achieve by diffusion alone, highlighting a significance in perfused oxygen-rich media [80]. While this μfluidic platform is capable of perfused cell culture, further optimization is needed to obtain *in vivo*-like vascular structures across device μchannels. Still, the μfluidic device is advantageous because there is no need for pumps, tubing, or auxiliary support components. The device’s compatibility with different cell phenotypes, multiplex and multiwell configuration, and adjustable flow conditions is essential for assessing biological and mechanical components that are necessary for a fully optimized liver model.

### 4.6. Future Research Directions

This study is limited with attention on APAP (having known metabolites), ASA (non-DILI), and NEF (DILI). Along with expanding assay panels of cell functions such as ATP (metabolic activity), albumin, urea, ROS (reactive oxygen), and BSEP (bile salt transporter), ongoing work will focus on different drug categories with the goal to broadly apply techniques for quick and easy identification of parent compound and toxic metabolite influencing. Future advancements will involve hepatic 3D spheroids that are formed within the device (i.e., no spheroid transfer) and complex arrangements of multi-organ tissues such as intestinal (upstream), hepatic (midstream), and target cancer (downstream) for body-on-chip systems.

## 5. Conclusions

The multiwell device, unidirectional flow, and six-sigma analysis presented here demonstrate the rapid detection of direct compound toxicity and trailing detection of cell-byproduct influencers across a cell culture platform. In trailing detections, the unmasking of cell-byproduct mechanisms allow for evaluations of metabolite-mediated toxicity through slow-, medium-, and fast-acting toxicity profiles, formerly concealed. With cell-byproduct secretions ascending from 0→subtoxic→borderline toxic→high toxic concentrations, this study explores real-time adaptive bioactivity trends and invites opportunities for cell-based screenings of new compounds, NCE, or to revisit market-removed drugs. Herein, lean six-sigma tactics are used to establish process control charts in the tracking of perpetually fluctuating cell health variances. Variance surveys of adjacent μchannel wells continuously evolve as cell-function indicators reveal upstream, midstream, and downstream mechanisms for process shifts (i.e., direct parent compound influence), special cause influencers (i.e., generated cell byproduct stimuli), or unusual outcomes (i.e., indirect secondary metabolite impacts). Akin to the idioms of precision medicine, personalized medicine, and predictive toxicology, bioreactors are progressing as fit-for-purpose *in vitro* tools providing complementary data intelligence.

We believe the patterns of observed dual toxicity (i.e., parent compound plus one or more cell byproducts) should be applicable across a wide variety of drugs, where cell metabolites are latent unknowns that may induce toxicity profiling.

## 6. Patents

Cell method and related systems for use in a fluidics device. US Pat No. 9,829,499.

## Supplementary Material

Supplementary Material

## Figures and Tables

**Table 1.** μFluidics Culture Device—Attributes and Infrastructure.

**Description**	96-well geometric footprint (½ area). 8 replicate flow channels (A-H) with linked wells.
External Dimensions:	Lab equipment agnostic. Compatible with most plate readers and most imagers.
Disposable Unit:	1 × use. Amendable application models such as modular cell-system configurations.
**Cell Alignment & Analysis**	Biologic support and functions.
Accessibility:	Cell harvesting (cellular analysis.). Media aspiration (fluid analysis).
Cell Phenotype:	Single cell phenotype. Multi-cell phenotypes. Cell-cell signaling.
Fluorescence Detection:	Optically clear wells. Image through plate bottom. Plate shall not auto-fluoresce.
**Format**	(x, y) well locations mimic traditional positioning. Z-height varies across 4 mm vertical.
Growth Area per Well:	Surface area is 0.167 cm^2^. 2D, flat surfaces. Side walls have edges for manufacturing.
Each Channel Includes:	Dose well (1); fluid regulator well (1); culture wells (9); waste well (1).
Device Composition:	Dosing wells (8); fluid regulator wells (8); culture wells (72); waste wells (8).
Z-Height Offset:	Wells 2–11, surfaces have 0.5 mm z-height offset amid adjacent (serially linked) wells.
**Channels for Fluid Flow**	Paths connect wells. Capillarity involves both 3-wall and 4-wall designs; air venting.
Fluid Traverse:	Passageway transport initiates in well 1 and terminates at well 12. Unidirectional flow.
Dose Well (Well 1):	Max vol. 500 μL. Fluid flow passageway from bottom of well 1 into top of well 2.
Regulator Well (Well 2):	Max vol. 100 μ.L. Force equilibrator w/fluid hydrodynamic. Fulcrum amid flow paths.
Culture Wells (Wells 3–11):	Working vol. of 30–100 μL; ½ area of 96-well format; 4 mm well height.
Sink Well (Well 12):	Max vol. 350 μL. Evaporation wick (syphon) pulls fluid from well 1 to well 12 (1-way).
Device Fluid Volume:	Per channel working volume is 1850 μL (1.85 mL). Per device (8 channels), 14.8 mL.
Fluidic Mixing in Wells:	Rapid mixing. Involves surface tension, laminar flow, well shapes, and fluid dispersion.
Flow Forces:	Gravity driven, hydrodynamics, capillarity, wicking.
**Materials in Fabrication**	Polystyrene, optically clear wells, conventional surface tension, base system with lid.
Thickness of Platform:	0.5 mm polymer thickness; parameter for injection molding and QA/QC non-warping.
Smoothness:	Well bottoms. Avoid bubbles in resin (injection mold flow rate). Contamination free.
**Manufacture Units**	Mass produced, 2 components, injection molding. Large quantity production capacity.
Manufacture Components:	Lid (1 per unit). Row cover (8 per unit). Wick (8 per unit).
Assembly of Units:	Ultrasound welding of molded parts. No glue. No adhesive. No bonding contaminants.
Surface Treatment:	Tissue culture plastic (TCP). Can be surface coated (e.g., proteins, non-binding, etc.).

**Table 2.** Coefficient of Variation for Intra-Plate Robustness.

Valid Obj Counts (Figure 7(a1,b1,c1))	Well 3	Well 7	Well 11
DMSO Vehicle Controls (Days 1, 3, 5)	(2.1%, 11.4%, 7.8%)	(2.6%, 13.0%, 0.64%)	(9.8%, 7.2%, 14.8%)
APAP (Days 1, 3, 5)	(2.8%, 20.1%, 12.8%)	(8.6%, 6.3%, 12.8%)	(1.9%, 8.2%, 5.8%)
NEF (Days 1, 3, 5)	(7.5%, 23.1%, 11.4%)	(3.3%, 5.8%, 0.04%)	(3.5%, 5.6%, 3.3%)
Tracing Fluid Flow Movements [23] (First Dose and Repeat Dosing)	Upstream (Wells 3–5)	Midstream (Wells 6–8)	Downstream (Wells 9–11)
≤1 h after each dose into well 1 (acute hydrodynamic forces in action)	not appraised	not appraised	not appraised
	SOP waits 1 h before analysis. Fnc of user interface (manual pipette dosing).		
1 h > dosing ≤ 3.3 h (transitional forces creating stability)	5–8%	5–6%	4–6%
>3.3 h till re-dosing (sustained equilibrium forces)	4–5%	4–5%	4–5%

**Table 3.** Trending VOC outcomes from direct parent compound and indirect cell byproducts.

Day	DMSO Mean UCL/LCL	APAP Correlations Superimposed over DMSO Data	NEF Correlations Superimposed over DMSO Data
1	6098.3 VOC 8173.8/4022.8 Figure 7(a1,a2)	All wells within ± 3σ boundaries	All wells within ± 3σ boundaries
		Conjecture: APAP concentrations and exposure time not yet influenced cells	Conjecture: NEF concentrations and exposure time not yet influenced cells
3	5265.7 VOC 6885.8/3645.6 Figure 7(b1,b2) “Time-delayed provocations”	Upstream wells 3–6 (2164.0,1393.5,2185.0,3260.0) below −3σ boundary Downstream wells7-11w/i ± 3σ boundaries	Upstream wells 3–4 (4445.0, 4756.0) w/i ± 3σ boundaries Midstream wells 5–8 (2300.0,356.0, 537.5, below −3σ boundary Downstream wells 9–11 (4374.0,5554.0,5926.5); w/i ± 3σ boundaries
		Conjecture: APAP w/direct negative influence at highest conc in upstream wells. APAP conc dilutions are less detrimental in downstream wells	Conjecture: NEF w/o direct upstream influence. Generating cell-byproducts w/negative influence in midstream wells 5–8 but not yet reached downstream wells 9–11
5	4353.2 VOC 5534.9/3171.4 Figure 7(c1,c2) “Time-delayed provocations”	All wells below −3σ boundary (889.0→2729.0)	Upstream wells 3–7 (4433.5→5109.0) w/i ±3σ boundaries. Observable distrubance at well 8 (17.0 VOC) with additional downstream wells 9–11 below −3σ boundary
		Conjecture: APAP w/direct negative influence at all ramped concentrations and time durations	Conjecture: NEF w/o upstream influence. Generating cell byproducts w/negative influence at downstream wells 8–11

**Table 4.** Summary of APAP-Metabolite variance from HepaRGs—generation and dissemination.

**APAP-GSH**	Highest MR @ 24 h	Most well-well Δ is 0.18 μM occurs at 24 h in midstream well 6 (Figure 9(a1)).
	Mean MR @ 24 h	Uniformwell-well Δ is measured in wells 4, 7, 8, and 9 (0.05 μM) (Figure 9(a1)).
	Negligeable MR @ 24 h	No well-well Δ found in downstream wells 10 and 11 (0.0 μM) (Figure 9(a1)).
	MR Compilations @ 24, 48, 72 h	24 h mean (0.06 μM) and the UCL (0.24 μM) are highest when compared alongside GSH-48 h and GSH-72 h; Figure 9(a1–a3). 48 h mean is least (0.042 μM) with UCL having a midrange distinction (0.125 μM); Figure 9(a2). 72 h me an is midtange (0.051 μM) with having the lowest distinction (0.11 μM) to divulge least variation Δ’s acros. deviae culture wells; Figure 9(a3).
	In Brief (Figure 9(a1–a3)):	3σ UCL is maximum at 24 h and decreases over time to indicate that cell generated APAP-GSH has the highest variance during early APAP exposure periods in upetream sites (wells 3–5; 0.05 μM; Figure 9(a1)) and conditions midstream culture wells with cell byproducts (weli 6; 0.18 μM).
**APAP-Sulfate**	Highest MR @ 72 h	Most well-well Δs are 12.79 μM and 11.11 μM occurring at 72 h in downstream wells 9 and 10 (Figure 9(b3)).
	Mean MR @ 72 h	Uniform well-well Δs are present in upstream wells 4–8 (0.84→4.9 μM) and downstream well 11 (0.9 μM) (Figure 9(b3)).
	Negligeable MR @ 24 h	Limited well-well Δ found in upstream wells 4 and 5 (~0.0 μM) (Figure 9(b1)).
	MR Compilations @ 24, 48, 72 h	24 h data mean is lowest (2.0 μM) with UCL having the lowest distinction (7.0 μM) to divulge least variation Δ’s across device culture wells; Figure 9(b1). ■48 h data mean is midrange (2.8 μM) with UCL also having a midrange distinction (9.0 μM); Figure 9(b2). 72 h mean (5.0 μM) and the UCL (18.5 μM) are the highest when compared alongside sulfate-24 h and sulfate-48 h; Figure 9b1–b3.
	In Brief (Figure 9(b1–b3)):	3σ UCL is minimum at 24 h and increases over time to indicate that cell-generated APAP-GSH has the highest variance at later APAP exposure periods with both upstream sites and prolonged exposure times augmenting downstream device sites with conditioned media; Figure 9b3 wells 9 and 10.
**APAP-GLU**	Highest MR @ 72 h	Most well-well variations are 68.1 μM & 48.6 μM occurring at 72 h in downstream wells 9 and 10 (Figure 9(c3)).
	Mean MR @ 72 h	Uniform well-well Δs are present in upstream wells 4–8 (0.91→21.24 μM) and downstream well 11 (3.9 μM) (Figure 9c3).
	Negligeable MR @ 24 h	Limited well-well Δ found in upstream wells 4 and 5 (~0.0 μM) (Figure 9(c1)).
	MR Compilations @ 24, 48, 72 h	24 h data mean is the lowest (9.0 μM) with UCL at the lowest distinction (30.0 μM) to divulge least variation Δs across device culture wells; Figure 9(c1). 48 h data mean is midrange (21.0 μM) with UCL also a midrange distinction (79.0 μM); Figure 9(c2). MRs also display notably high variations in culture wells 8 and 9 (63.5 μM and 34.7 μM). 72 h mean (23.0 μM) and the UCL (92.0 μM) are the highest when compared alongside Glu-24 h and Glu-48 h; Figure 9(c1–c3).
	In Brief (Figure 9(c1–c3)):	3σ UCL is minimum at 24 h and increases over time to indicate that cell-generated APAP-Glu has high variance at both intermediate and late APAP exposure periods (Figure 9(c2) wells 8–9 and Figure 9(c3) wells 9–10).

**Table 5.** Trending relevance displays in Figures 10–12.

Rows	Purpose	Description
**a1→d1**	Reveal cell functions having discrete statistical analysis, i.e., *Generation of Data*. The vertical axis are fold-changes related to DMSO vehicle control.	DMSO vehicle studies (white bars) are normalized at 1, i.e., 100% normal function per well; the compound-of-interest data (black bars) are function fold-changes versus DMSO controls.
**a2→d2** (system)	Re-expressions of data using X control charts, continuous systems (e.g., *fluidics assembly line*), to identify DMSO control averages (Mean) and ±3σ DMSO offsets (UCL and LCL). The vertical axis are fold-changes related to DMSO vehicle control.	Superimposed over DMSO controls are compound-of-interest results to reveal statistical shifts in cell bioactivity along upstream (wells 3–5), midstream (wells 6–8), and downstream (wells 9–11) cell stations.
**a3→d3** (adjacent)	Display adjacent-well variations using MR control charts, i.e., *Application to Diagnose Cell-Generated Stimuli*. The vertical axis is fold-changed to probe well-well irregularities, magnitudes are absolute values, orientation with DMSO vehicle = 1 (line shifts @ 1). Standard deviations are +1σ, +2σ, +3σ.	Blending of system trends with adjacent-well analysis facilitate deductions for direct drug influence (i.e., process shift), indirect drug influence (i.e., special cause (cell generated secretions)), and indirect tertiary influence (i.e., unusual outcomes (secondary cell metabolite)).

**Table 6.** Summary of LDH indicator responses.

**Study 1** ASA on HepaRG cells	Trending Chart (system wide):	Discloses a cyclical culture response across the fluidic channel with most datapoints inside ± 3σ DMSO boundaries, Figure 10(a2).
	Variability Chart (adjacent well):	Shows well 4 is differentiable from wells 5–11 (1.6-fold vs. 0.9–1.35-fold), Figure 10(a3), yet the adjacent-well asymmetry is negligible as determined “ns” in Figure 10(a1).
	Blended Data (Figure 10(a1–a3)):	Infers ASA does not induce LDH release or incite hepatoxicity in HepaRG cells.
**Study 2** ASA on Rat Heps	Trending Chart (system wide):	Discloses an inverted bell curve response, Figure 10(b2), revealing rat hepatocytes have greater activity above HepaRG (1.35-to-2.2-fold vs. 0.5-to-1.3-fold). The rat hepatocytes up-, mid-, and downstream cell functions are tempered (i.e., fewer cyclical fluctuations) compared with HepaRG cells, Figure 10(b2) vs. Figure 10(a2).
	Variability Chart (adjacent well):	Confirms irregular functions at upstream well 4 (i.e., 2.4-fold change) implying direct ASA exposure induces a process shift. Moreover, irregularity in downstream wells 10–11 infer indirect ASA exposures have special cause implications (e.g., cell generating impacts) as midstream wells have lower functions; Figure 10(b2,b3).
	Blended Data (Figure 10(b1–b3)):	Infers ASA-induced endogenous metabolites stimulate LDH release in downstream rat hepatocytes
**Study 3** NEF on HepaRG cells	Trending Chart (system wide):	Discloses upstream activity with fold changes 1.4 to 2.6 above DMSO controls, with cyclical data patterns; Figure 10(c2).
	Variability Chart (adjacent well):	Confirms irregularities in upstream wells 4–5 (1.45 and 2.1-fold Δ) and midstream wells 6–7 (1.8 and 2.2-fold A); Figure 10(c3).
	Blended Data (Figure 10(c1–c3)):	Infers NEF directly induces an upstream process shift in HepaRG cells (wells 3–6); i.e., direct NEF influence.
**Study 4** ASA on Rat Heps	Trending Chart (system wide):	Discloses three cell-function peaks to at upstream well 3 (3.1-fold Δ), midstream well 7 (2.3-fold Δ), and downstream well 10 (2.4-fold Δ) that are interspersed between normal cell-function outcomes; Figure 10(d2).
	Variability Chart (adjacent well):	Confirms irregularities in upstream well 4 (i.e., process shift = direct NEF influence), midstream wells 7–8 (i.e., special cause, cell secretions, or generated metabolite), and downstream wells 10–11 (i.e., unusual outcome or tertiary/secondary metabolite = indirect NEF influence); Figure 10(d3).
	Blended Data (Figure 10(d1–d3)):	Infers NEF directly, indirectly, and tertiary stimulate LDH release in rat heps.

**Table 7.** Summary of CYP3A4 indicator responses.

**Study 5** ASA on HepaRG cells	Trending Chart (system wide):	Discloses a bell curve response having inferior function at upstream well 3 (0.4-fold Δ), maximum functions in midstream wells 6–9 (1.3-fold Δ), and diminishing function at downstream well 11 (0.7-fold Δ); Figure 11(a2).
	Variability Chart (adjacent well):	Confirms irregularities in upstream well 4 (process shift = direct ASA influence), and midstream wells 6–9 (special cause or generated metabolite = indirect ASA influence) given that well 5 has lower functions; Figure 11(a3).
	Blended Data (Figure 11(a1–a3)):	Infers ASA directly and indirectly stimulate CYP3A4 activity in HepaRG cells.
**Study 6** ASA on Rat Heps	Trending Chart (system wide):	Discloses an exponential growth curve response, Figure 11(b2), revealing rat hepatocyte CYP3A4 functions 7.5 × to 33.1 × are higher than HepaRG functions, ranges being 3.0-to-43.0 fold versus 0.4-to-1.3 fold, respectively.
	Variability Chart (adjacent well):	Confirms irregularities having partitioned influences. Influence-1, well 3, being unchanged and replicates DMSO control functions (i.e., no direct ASA influence = no process shift). Influence-2, wells 4–9, have upregulated CYP3A4 at 1.5-to-7.0-fold ranges (i.e., special cause A (e.g., metabolite A = indirect ASA influence)). Influence-3, wells 10–11, have top-heavy upregulated CYP3A4 with 20-to-40-fold ranges (i.e., special cause A↑ (e.g., additional cell generating impacts = indirect ASA influence)); Figure 11(b3).
	Blended Data (Figure 11(b1–b3)):	Infers ASA endogenous metabolites stimulate CYP3A4 induction in rat hepatocytes, i.e., wells 4–11.
**Study 7** NEF on HepaRG cells	Trending Chart (system wide):	Discloses NEF outcomes with fold levels 0.9–1.0-fold below the DMSO vehicle control. A marginal rise is evident at wells 7–8; Figure 11(c2).
	Variability Chart (adjacent well):	Confirms activity as minimal, no irregularity across the entire μchannel (i.e., a process shift as direct NEF exposure inhibits CYP3A4 functions); Figure 11(c3).
	Blended Data (Figure 11(c1–c3)):	Infers that NEF directly impedes CYP3A4 in HepaRG cells.
**Study 8** ASA on Rat Heps	Trending Chart (system wide):	Discloses three notable cell-function influencers; Influence-1 with upstream wells 3–6 at fold levels 0.6–1.0 below the DMSO vehicle control, Influence-2 shows wells 9–10 at fold levels 2.3-to-8.3-fold above DMSO control, Influence-3 displays downstream well 11 at 1.9-fold above the DMSO control; Figure 11(d2).
	Variability Chart (adjacent well):	Confirms irregularities in upstream wells 4–6 (i.e., process shift = direct NEF influence), highly irregular in downstream wells 9–10 (i.e., special cause or generated metabolite = indirect NEF influence), and a decline in downstream well 11 (i.e., unusual outcome = tertiary indirect NEF influence); Figure 11(d3).
	Blended Data (Figure 11(d1–d3)):	Infers NEF has direct, indirect, and tertiary influences on CYP3A4 mutable responses in rat hepatocytes.

**Table 8.** Summary of GSH indicator responses.

**Study 9** ASA on HepaRG cells	Trending Chart (systemwide):	Discloses a delayed exponential growth curve with inferior functions upstream and midstream (0.2–0.6-fold Δ) and ramping at downstream well 11 (1.19-fold Δ). Only well 11 resides in ±3σ DMSO boundary limits; Figure 12(a2).
	Variability Chart (adjacentwell):	Confirms functions remain similar across the entire μchannel (not well 11), inferring direct ASA exposure induces a process shift; Figure 12(a3).
	Blended Data (Figure 12(a1–a3)):	Infers ASA directly impedes GSH in HepaRG cells.
**Study 10** ASA on Rat Heps	Trending Chart (system wide):	Disclose an atypical-inverted bell curve response with upstream increases (4.5 to 5.8-fold), midstream DMSO vehicle levels at 1.0, a 2nd midstream increase (3.0 to 9.0-fold), and ramping downstream activity at 15.0 to 26.5-fold Δ’s; Figure 12b2.
	Variability Chart (adjacent well):	Confirms functions are similar in upstream wells 3–5 (process shift = direct ASA influence), then upregulated in midstream wells 7–9 (3.4 to 7.0-fold) occurring after non-significant well 6, (indirect ASA influence = special cause A (e.g., metabolite A)), then top-heavy upregulation in wells 10–11 (10.9 to 18.4-fold), i.e., indirect ASA influence = special cause A↑ (e.g., additional cell generating impacts); Figure 12(b3).
	Blended Data (Figure 12(b1–b3)):	Infers ASA metabolites stimulate GSH induction in rat hepatocytes (wells 7–11).
**Study 11** NEF on HepaRG cells	Trending Chart (system wide):	Discloses a bell curve response having upstream wells below the DMSO LCL (−3σ), well 8 approaching DMSO control at 1, and downstream wells below the DMSO LCL (−3σ); Figure 12(c2).
	Variability Chart (adjacent well):	Confirms functions are similar in upstream wells 4–7 (process shift = direct NEF influence) and similar in downstream wells 9–11 (special cause or generated metabolite = indirect NEF influence); Figure 12(c3).
	Blended Data (Figure 12(c1–c3)):	Infers that NEF directly and indirectly impedes GSH in HepaRG cells.
**Study 12** ASA on Rat Heps	Trending Chart (system wide):	Discloses an inverted bell curve response having segmented influencers; Influence-1 in upstream wells 3–5 at fold levels 2.0–2.5 above the DMSO vehicle control; Influence-2 in wells 8–10 at fold levels 2.0–7.0 above DMSO controls, Influence-3 in downstream well 11 displaying fold-level 0.1 below the DMSO control; Figure 12(d2).
	Variability Chart (adjacent well):	Confirms functions are similar in upstream wells 3–5 (process shift = direct NEF influence), returns to DMSO levels at well 7, becomes upregulated in downstream wells 8–10 (special cause or generated metabolite = indirect NEF influence), then experiences an irregularity in downstream well 11 (infers an unusual outcome); Figure 12(d3).
	Blended Data (Figure 12(d1–d3)):	Infers NEF directly, indirectly, and tertiary stimulate GSH mutable responses in rat hepatocytes.

**Table 9.** Summary of trending responses and predictable exposure stimuli using cell-based screening indicators LDH, CYP3A4, and GSH. Coalesced cell function indicator data from Figures 10–12.

Study	Feature			{—–Upstream—–} Device Culture Wells {–Downstream–}								
	Cell Phenotype	Exposure Drug	Cell Function	3	4	5	6	7	8	9	10	11
Ctrl	ALL	DMSO	ALL	DMSO vehicle control: 0.1% dimethyl sulfoxide (labeled as 0.1 *v/v*) Establishing baseline and standard cell culture limits = Normalized at 1								
1	HepaRG	ASA	LDH	w/i control limits (i.e., not significant [ns] compared with the DMSO vehicle)								
3	HepaRG	NEF	LDH	Process Shift (↑fnc)				w/i Control Limits				
5	HepaRG	ASA	CYP3A4	Process Shift (↓fnc)	w/i Control Limits		Special Cause (* minimal ↑fnc)				w/i Control Limits	
7	HepaRG	NEF	CYP3A4	Process Shift (↓fnc)								
9	HepaRG	ASA	GSH	Process Shift (↓fnc)								w/i Control Limits
11	HepaRG	NEF	GSH	Process Shift (↓fnc)					w/i Control Limits	Special Cause (*minimal ↓fnc)		
2	Rat Hep	ASA	LDH	Process Shift (↑fnc)	w/i Control Limits						Special Cause (↑fnc)	
4	Rat Hep	NEF	LDH	Process Shift (↑fnc)	w/i Control Limits			Special Cause (↑fnc)	w/i Control Limits		Unusual Outcome (↑fnc)	
6	Rat Hep	ASA	CYP3A4	w/i Control Limits	Special Cause (A); (↑fnc)						Special Cause (A+); (↑fnc)	
8	Rat Hep	NEF	CYP3A4	Process Shift (↓fnc)				w/i Control Limits		Special Cause (↑fnc)		Unusual Outcome (↓fnc)
10	Rat Hep	ASA	GSH	Process Shift (↑fnc)			w/i Control Limits	Special Cause (A); (↑fnc)			Special Cause (A↑); (↑fnc)	
12	Rat Hep	NEF	GSH	Process Shift (↑fnc)			w/i Control Limits		Special Cause (↑fnc)			Unusual Outcome (↓fnc)

Nomenclature: **Process Shift**= direct cell culture reaction to drug. **Special Cause**= indirect cell culture reaction to drug (e.g., cellular byproduct/metabolite). (A) and (A↑) and (A+) = consecutive, albeit distinct, ramps of special causes. **Unusual Outcome**= a secondary indirect cell culture reaction occurring after an initial special cause activity (e.g., tertiary or secondary cellular byproduct/metabolite). **w/i Control Limits**= no significant changes when contrasted against DMSO vehicle control data. ↓ = Decrease. ↑ = Increase. * = Statistical relevance (Figures 10–12).

**Table 10.** μFluidic contemporary technologies with fit-for-purpose application.

Company	Device	Transport	Microenvironment
BellBrook	IUVO	Cell movement through ECM	Micro conduits for invasion cell analysis across 3D environments
BioIVT	HepatoPac	Static co-cultures	Liver modules that are based culture patterning for segmented hepatocytes and stromal cells.
CNBio	Microphysiologic Platforms	Mixing chamber and recirculating flow	Scaffolds for 3D tissue formation and transwells for basal component re-circulation
Emulate	Organ on Chips	Molecular scaffolds in microchannels	Suite of organ-chip models with automated flow, instruments, consumables, and software; microfluidic channels lined with cells
Hurel	Hurelflow	Recirculating	Three devices on a biochip. Each device with two cell compartments aligning co-cultures with reactive metabolites
InSphero	Akura Platforms	Gravity driven and rocker	InSight microtissue models in 3D for co-culture liver cells. Microslides, robotics, and cell spheroid pipette transfers
Kirkstall	Quasi Vivo	Recirculating	Interconnected flows, peristaltic pump, chambers for cultures, tubes for media transports, and Lego-like building system.
Millipore	CellAsic ONIX2	Flow gradients	Automated platform, manipulation of culture parameters, and continuous culture observation
Mimetas	Organoplate	Gravity driven	Perfusable vascular liver models with Phaseguide meniscus channels and barriers for chemical and compound gradients
TissUse	Human on Chip	Micropump chips and dynamic circulations	To simulate activity of multiple connected human organs; systemic.

## Appendix A

### Appendix A.1. Terminology to Define Modulation Traits of Cell Data for Process Shift, Special Cause, and Unusual Outcome

The terms process shift, special cause, and unusual outcomes represent six-sigma expressions across a unidirectional manufacture production line that is based on (i) in-line sequential workstations, (ii) resultant product variability outcomes from each workstation that is doing an itemized job, and (iii) the final product at the end of the production line. When a variation falls out of ±3σ boundaries, per workstation, an indexed terminology is rendered based on physical production-line location as related to upstream, midstream, or downstream worksites. Associatively, for SsWaterfall, a single platform channel involves 1-way flow and has nine cell culture workstations (wells 3–11). Each worksite accomplishes itemized functions in response to station-specific exposures allied with timelines, concentration ramps, and specific compound traits. Unidirectional flow infers that the downstream distribution of drugs remains as both conclusive and observable elements, unmixed (unblended), as sample traits that are critical for assay evaluations. Herein, a process shift is defined as a direct drug influence (Table 5, row a3–d3, Description), where modulations are directly attributed to the dosing compound, are usually found close to the dosing well, and have inflection cadences confirmed against DMSO controls. A special cause is also a modulation with two-fold comparisons against an upstream process shift and well-specific DMSO controls. The change is not directly associated with the dosing compound. Instead, an indirect association can be represented as a cell byproduct that induces a downstream culture site modulation (Tables 6 and 7, Studies 2, 4, 5, 6, and 8 variability charts). The physical location is important as upstream cell-generated byproducts are needed to influence downstream workstation functions. Accordingly, the special cause is inferred from a 1st cell-generated byproduct. Similarly, an unusual outcome is also a modulation, but the change is from a 2nd cell-generated byproduct to induce worksite inflections occurring after a special cause (Table 8, Studies 10, 11, 12 Variability Charts).

### Appendix A.2. Modulation of Cell Byproducts/Metabolites

*In vivo*, particular hepatic toxicities emerge as native liver cells generate cell secretions that can catalyze liver damage. The advancement of *in vitro* models that better mimic behaviors of native liver tissue remains an industry challenge. To enrich scientific understanding, *in vitro* culture systems may benefit from established cell–cell networks and the intrinsic interactions between a parent compound (upstream direct effect) and the maturity of cell-generated secretions (downstream indirect effect). In-line arrangements of cell cultures potentially include:

- Hepatocyte → hepatocyte → hepatocyte → (channel multiwells 3–9 as presented in the current text);
- Liver zone-1 (wells 3–5) → liver zone-2 (wells 6–8) → liver zone-3 (wells 9–11); correlated to cell maturity;
- Intestinal (wells 3–4) → liver (wells 5–9) → target cancer (wells 10–11); correlated to multiorgan systems, etc.

## Appendix B

### Trending Effects of Vehicle DMSO Used for Normalization across Wells

Charts are normalized to facilitate well-to-well data comparisons. The technique for normalization is visualized in Figure A1 as evaluated for assays LDH (U/L), GSH (μM), and CYP3A4 (RLU). Panels (a1, b1, c1) are DMSO-only controls displaying 3 study replicates along with the combined average. Panels (a2, b2, c2) are the same DMSO controls compared against an alternative compound, norbuprenorphine at 20 nM dose, to illustrate variability in replicate drug studies and variability against DMSO controls. Panels (a3, b3, c3) are the same DMSO controls compared against three alternative compounds, (flurbiprofen at 573 μM, norbuprenorphine at 20 nM, propranlol at 2 μM) to signify variability amid exposure drugs.
