# Supplementary material for: Rapid Detection of Direct Compound Toxicity and Trailing Detection of Indirect Cell Metabolite Toxicity in a 96-Well Fluidic Culture Device for Cell-Based Screening Environments: Tactics in Six Sigma Quality Control Charts
Source: Appl Sci (Basel). Author manuscript; Available in PMC 2023 Jul 27. (PMC10374175; doi:10.3390/app12062786)
Supplement: Supplementary Material [file NIHMS1857829-supplement-Supplementary_Material.pdf]

applied sciences

MDPI

## Supplementary Materials

### S.1. *Trending effects of DMSO Vehicle vs Exposure Compounds*

To demonstrate cell culture modulations ranging from raw data acquisition and into DMSO normalizations, HepaRGs were seeded as described in Section 2.3.1 using ancillary compounds Phenacetin, Mebendazole, Propranlol as described in SOP Sections 2.4 and 2.9. Cells were washed with phosphate-buffered saline (PBS) and exposed to 0.1%DMSO (vehicle-control) medium containing the desired concentration of 0.134 mM Phenacetin, 1.26 μM Mebendazole, and 2 μM Propranlol into dose well 1, for 7 Days.

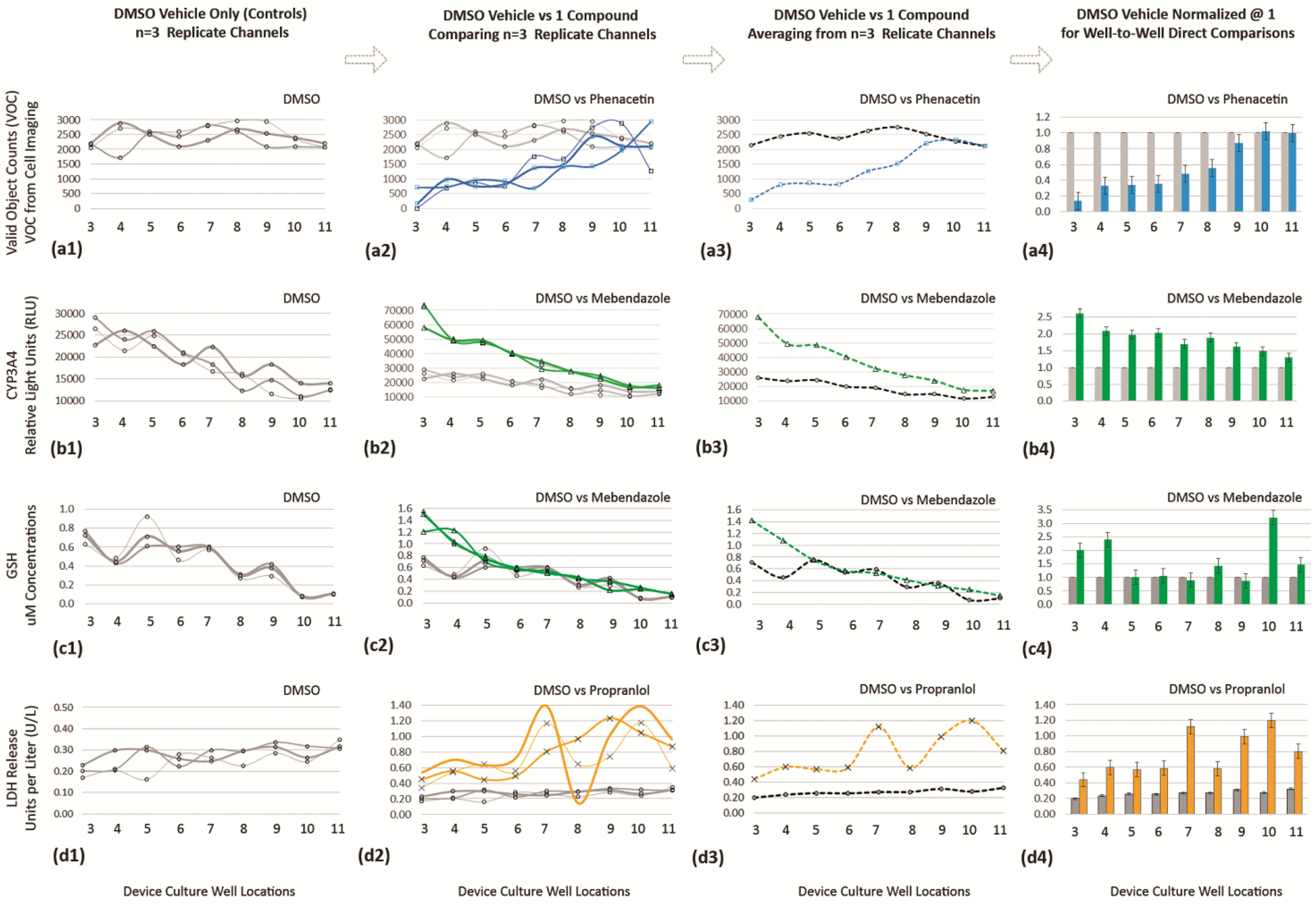


**Figure S1.** Surveillance of trending variability across device multiwell locations 3-11 (n=3), observed at day 7. (**a1**–**a4**) VOC as differentiated by microscopy and quantity count. (**b1**–**b4**) CYP3A4 as dif-ferentiated by cell function and RLU. (**c1**–**c4**) GSH as differentiated by cell function and μM concen-trations. (**d1**–**d4**) LDH Release as differentiated by cell function and units per liter (U/L). All line-graphs are individual experiments corresponding to one channel having 9 linked multiwells.

#### S.1.1. DMSO Vehicle Controls at Day 7

DMSO controls are 0.1%DMSO (vehicle-control) medium corresponding to the com-pound of interest (Figure S1(a1,b1,c1,d1)). This concentration is dosed into well 1, with fluidic channels facilitating flow of compound across multiwells while establishing auto-concentration gradients across the row from well-1 to well-12 [23]. Since the concentration is different at each culture site over time (Figure 4(b1,b2, b3)), then each multiwell in the μchannel can be considered a distinct experiment with unalike flow dynamics across

concentration and time. (a1) Illustrates VOCs retaining equivalent numbers across all multiwells. (b1,c1) Illustrates decreasing CYP3A4 and GSH activity in downstream wells (i.e., wells having less DMSO exposures). (d1) Illustrates LDH Release remaining consistent across all multiwells.

S.1.2. DMSO Vehicle vs Phenacetin at Day 7

Phenacetin is dosed at 10x clinical Cmax as dilution curves quickly decrease exposure concentrations across the channel multiwells (Figure S1(a2,a3,a4)). (a2) Phenacetin displays lower upstream VOCs indicating cells are waning from the culture surface at higher concentrations. (a3) Averaged DMSO vs Phenacetin studies (n = 3). (a4) To account for culture variance based on changing DMSO exposures, cultures are normalized with DMSO study outcomes=1, allowing well-well comparisons of drug exposures.

S.1.3. DMSO Vehicle vs Mebendazole at Day 7

Mebendazole is dosed at 10x clinical Cmax as dilution curves quickly decrease expo-sure concentrations across the channel multiwells (Figure S1(b2,b3,b4)). (b2) Meben-dazole displays higher CYP3A4 across all channel multiwells, indicating more CYP3A4 function with higher compound exposures. (b3) Averaged DMSO vs Mebendazole studies (n = 3). (b4) To account for culture variance based on changing DMSO exposures, cultures are normalized with DMSO study outcomes=1, allowing well-well comparisons of drug exposures.

S.1.4. DMSO Vehicle vs Mebendazole at Day 7

Mebendazole is dosed at 10x clinical Cmax as dilution curves quickly decrease expo-sure concentrations across the channel multiwells (Figure S1(c2,c3,c4)). (b2) Mebendazole displays higher GSH in upstream wells 3-4 before equating with DMSO function out-comes, indicating more GSH function with acute higher compound exposures. (b3) Aver-aged DMSO vs Mebendazole studies (n = 3). (b4) To account for culture variance based on changing DMSO exposures, cultures are normalized with DMSO study outcomes=1, al-lowing well-well comparisons of drug exposures.

- Analysis Prudence (c4: DMSO Normalization): The y-axis range, for values below 1, may influence the normalization outcomes as equations with small denominators can exaggerate conclusions. For example, the GSH graph (c3) displays accurate upstream GSH differences, wells 3-4, as downstream wells 5-11 retain similar responses to DMSO controls. Conversely, for the same data, GSH normalized graph (c4) displays significant downstream differences in wells 10-11, an imprecise conclusion induced by small denominator values. To avert this consequence, raw experimental values such as sizeable RLU outcomes can be used to normalize data, prior to conversion into drug concentration specifics (e.g., (b1-b4) CYP3A4 analysis). Likewise, biological replicates are performed using two-way ANOVA tests to assess significance of responses, * $p<0.05$, ** $p<0.01$, *** $p<0.001$, ****$p<0.0001$; ns: not significant, as displayed in Figures 10 a1-d1, 11 a1-d1, and 12 a1-d1.

S.1.5. DMSO Vehicle vs Propranlol at Day 7

Propranlol is dosed at 10x clinical Cmax as dilution curves quickly decrease exposure concentrations across the channel multiwells (Figure S1(d2,d3,d4)). (d2) Propranlol dis-plays high LDH Release upstream, wells 3-6, then fluctuating high/low release, wells 7-11, to indicate mutable cell functions with potential cell-byproduct influencers, wells 7-11. (d3) Averaged DMSO vs Propranlol studies (n = 3). (b4) To account for culture variance based on changing DMSO exposures, cultures are normalized with DMSO study outcomes=1, allowing well-well comparisons of drug exposures.
